# Attenuation of HOIL-1L ligase activity promotes systemic autoimmune disorders by augmenting linear ubiquitin signaling

**DOI:** 10.1172/jci.insight.171108

**Published:** 2024-02-08

**Authors:** Yasuhiro Fuseya, Keiichiro Kadoba, Xiaoxi Liu, Hiroyuki Suetsugu, Takeshi Iwasaki, Koichiro Ohmura, Takayuki Sumida, Yuta Kochi, Akio Morinobu, Chikashi Terao, Kazuhiro Iwai

**Affiliations:** 1 Department of Molecular and Cellular Physiology and; 2 Department of Rheumatology and Clinical Immunology, Graduate School of Medicine, Kyoto University, Kyoto, Japan.; 3 Laboratory for Statistical and Translational Genetics, RIKEN Center for Integrative Medical Sciences, Yokohama, Japan.; 4 Department of Orthopaedic Surgery, Graduate School of Medical Sciences, Kyushu University, Fukuoka, Japan.; 5 Department of Rheumatology and Clinical Immunology, Kobe City Medical Center General Hospital, Kobe, Japan.; 6 Department of Rheumatology, Faculty of Medicine, University of Tsukuba, Tsukuba, Ibaraki, Japan.; 7 Department of Genomic Function and Diversity, Medical Research Institute, Tokyo Medical and Dental University, Tokyo, Japan.; 8 Laboratory for Autoimmune Diseases, RIKEN Center for Integrative Medical Sciences, Yokohama, Kanagawa, Japan.; 9 Clinical Research Center, Shizuoka General Hospital, Shizuoka, Japan.; 10 Department of Applied Genetics, School of Pharmaceutical Sciences, University of Shizuoka, Shizuoka, Japan.

**Keywords:** Autoimmunity, Cell Biology, Autoimmune diseases, Lupus

## Abstract

Linear ubiquitin chains, which are generated specifically by the linear ubiquitin assembly complex (LUBAC) ubiquitin ligase, play crucial roles in immune signaling, including NF-κB activation. LUBAC comprises catalytic large isoform of heme-oxidized iron regulatory protein 2 ubiquitin ligase 1 (HOIL-1L) interacting protein (HOIP), accessory HOIL-1L, and SHANK-associated RH domain-interacting protein (SHARPIN). Deletion of the ubiquitin ligase activity of HOIL-1L, an accessory ligase of LUBAC, augments LUBAC functions by enhancing LUBAC-mediated linear ubiquitination, which is catalyzed by HOIP. Here, we show that HOIL-1L ΔRING1 mice, which exhibit augmented LUBAC functions upon loss of the HOIL-1L ligase, developed systemic lupus erythematosus (SLE) and Sjögren’s syndrome in a female-dominant fashion. Augmented LUBAC activity led to hyperactivation of both lymphoid and myeloid cells. In line with the findings in mice, we sought to identify missense single nucleotide polymorphisms/variations of the *RBCK1/HOIL-1L* gene in humans that attenuate HOIL-1L ligase activity. We found that the R464H variant, which is encoded by rs774507518 within the *RBCK1/HOIL-1L* gene, attenuated HOIL-1L ligase activity and augmented LUBAC-mediated immune signaling, including that mediated by Toll-like receptors. We also found that rs774507518 was enriched significantly in patients with SLE, strongly suggesting that *RBCK1/HOIL-1L* is an SLE susceptibility gene and that augmented linear ubiquitin signaling generated specifically by LUBAC underlies the pathogenesis of this prototype systemic autoimmune disease.

## Abstract 2

<p align=”left”>Rare<em>&nbsp;RBCK1/HOIL-1L</em>&nbsp;variant&nbsp;is a systemic lupus erythematosus susceptibility gene that augments&nbsp;linear ubiquitin assembly complex&nbsp;function via attenuation of HOIL-1L ligase.</p>

## Graphical abstract

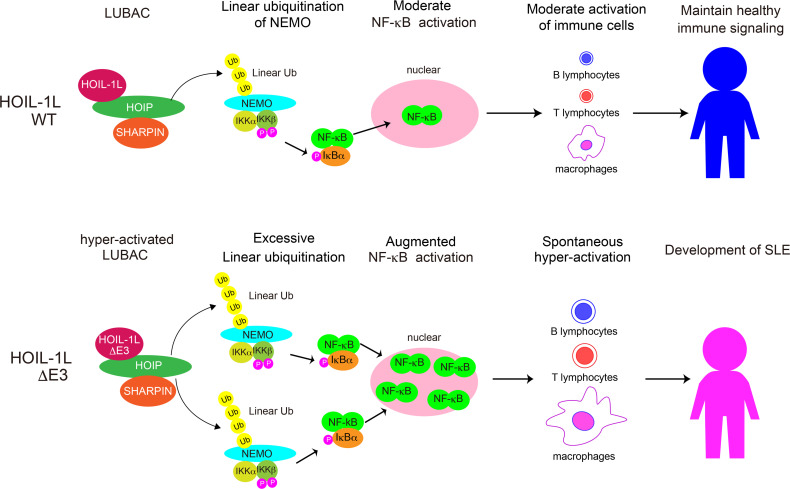

## Introduction

Systemic lupus erythematosus (SLE) is a life-threatening systemic autoimmune disease characterized by production of autoantibodies induced by dysregulated functioning of the acquired and innate immune systems (1–3). Multiple organs, including the kidney, skin, and central nervous system, are affected, with potentially severe consequences. Also, SLE is often comorbid with other autoimmune disorders, such as Sjögren’s syndrome (SS), which is characterized by dry eyes and dry mouth (“sicca symptoms”) (1–5). Both SLE and SS exhibit a striking female predominance. Advances in corticosteroids, immunosuppressive agents, and other therapies have helped control the activity of SLE, and life expectancy and quality of life have improved (1). However, existing treatments, including immunosuppressive therapy, can cause serious treatment-related comorbidities, such as infections, renal impairment, metabolic syndrome, and atherosclerosis (1–3). Thus, elucidation of the pathophysiology of SLE, and further development of brand-new therapeutic drugs, are awaited.

The pathophysiology of SLE is still largely unknown; however, we do know that both genetic and environmental factors play a central role (1). Genome-wide association studies (GWAS) using single nucleotide polymorphisms (SNPs) have identified more than 150 susceptibility loci relevant to SLE. Accumulating human genetic evidence, together with transcriptional studies and mouse models, support a critical role for nucleic acid sensors, (including TLRs), lymphocyte signaling molecules such as NF-κB, and IFN production pathways in the pathogenesis of SLE (3). These pathways interact with each other; for example, stimulation of plasmacytoid dendritic cells via TLR7 and -9 leads to activation of NF-κB and suppresses the inhibitory effects of glucocorticoids on the IFN pathway (6). Multiple genetic loci suggest that multiple activating molecules reside upstream of NF-κB; these include *LRRK1* (7), tumor necrosis factor-α–induced protein 3 (*TNFAIP3*) (8), and *TNFAIP3* interacting protein 1 (*TNIP1*) (9). However, the detailed mechanisms underlying interactions between these upstream molecules and NF-κB are unclear in the context of SLE (10).

The *TNIP1* risk allele reduces expression of the TNIP1 protein, also known as A20-binding inhibitor of NF-κB (ABIN1), a ubiquitin-binding protein (11, 12). ABIN1 interacts specifically with linear ubiquitin chains through its ubiquitin-binding domain, which is involved in signaling but not degradation (13). The linear ubiquitin chains, which are generated specifically by the linear ubiquitin assembly complex (LUBAC) ubiquitin ligase in response to various stimuli, including TLRs, play crucial roles in NF-κB activation and protection from cell death (14–16). Recently, we identified a potential mechanism by which attenuated expression of ABIN1 leads to NF-κB activation, pointing to the importance of regulating linear ubiquitin chains in SLE (13). ABIN1 attenuates linear ubiquitin chain signaling elicited TLR ligands by delivering components of the signaling proteins to lysosomes for degradation via autophagy in a linear ubiquitination-dependent manner (13). Consistent with findings in humans, mice expressing an ABIN1 mutant defective in ubiquitin binding, as well as ABIN1-null mice, exhibit lupus-prone phenotypes, including glomerulonephritis and generation of autoantibodies (12, 17–19). These data suggest that enhanced linear ubiquitin chain signaling leads to NF-κB activation and development of SLE.

**Figure 1:**
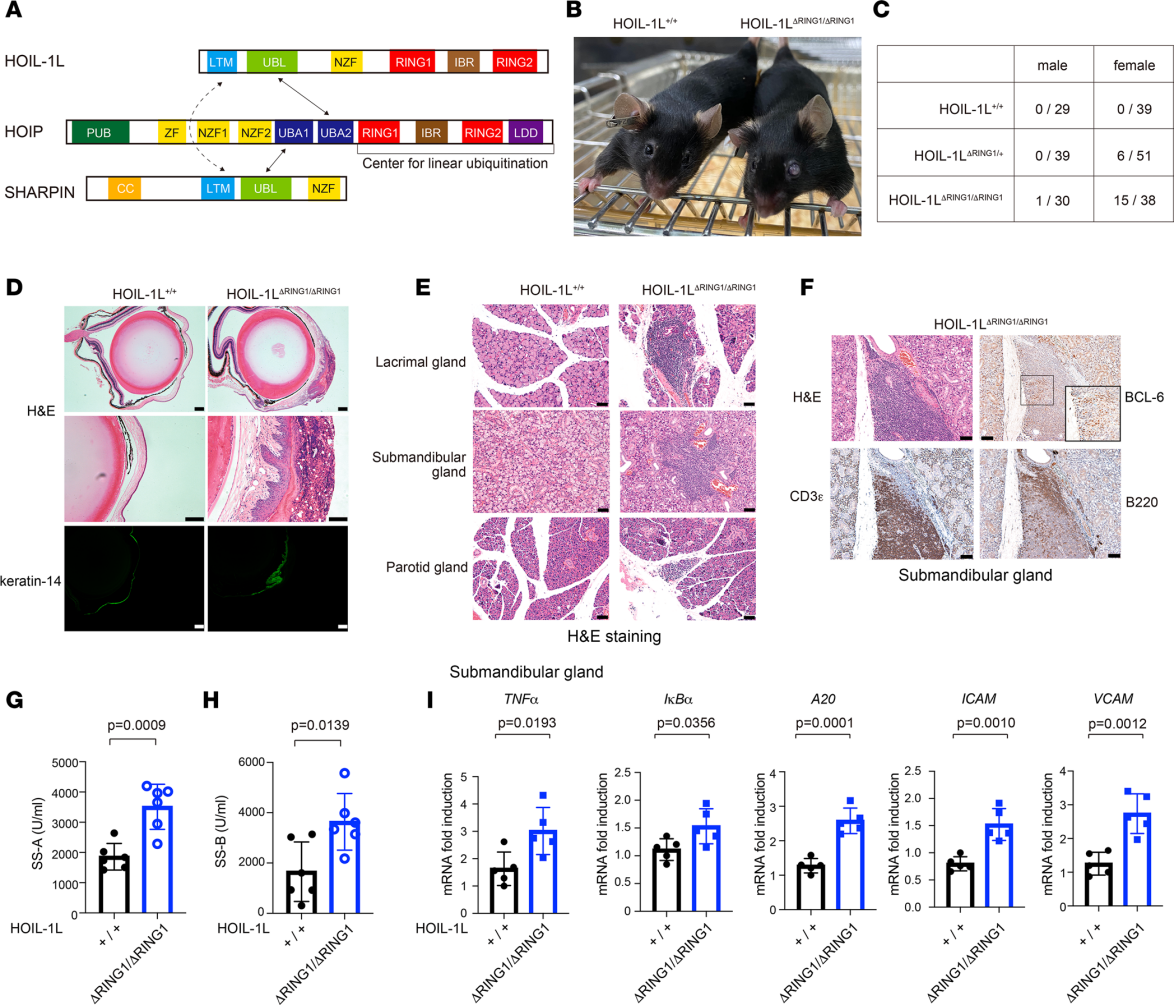
HOIL-1L ΔRING1 mice show SS-like symptoms. (**A**) Schematic representation of the LUBAC subunit domains. (**B**) Macroscopic pictures of littermate female mice of the indicated genotypes (aged 7 months). (**C**) Numerator: number of mice of the indicated genotype that exhibited opaque plaques on the cornea at 7–12 months of age. Denominator: total number of mice of the indicated genotype at 7–12 months of age. (**D**) Histological analysis, performed by H&E staining and immunostaining of keratin-14 in eye sections from 10-month-old littermate female mice of the indicated genotype. Scale bars, 200 µm. (**E**) H&E staining of lacrimal, submandibular, and parotid glands from littermate female mice of the indicated genotype (7 months of age). Scale bars, 50 µm. (**F**) Histological analysis, by H&E staining and immunostaining, of B220, CD3ε, and BCL-6 expression in the submandibular glands of 7-month-old littermate female mice of the indicated genotype. Scale bars, 50 µm. Inset, 2× magnification of background image. (**G** and **H**) Levels of anti–SS-A (**G**) and anti–SS-B (**H**) antibodies in the serum from 7- to 10-month-old littermate female mice of the indicated genotype. Data are expressed as the mean (*n* = 6, each genotype) ± SD. (**I**) Quantitative PCR (qPCR) analysis of submandibular glands from female mice (aged 6–7 months) of the indicated genotype. Data are expressed as the mean (*n* = 5, per genotype) ± SD. (**G**–**I**) *P* values were calculated using a 2-tailed Student’s *t* test.

LUBAC comprises 3 subunits: catalytic large isoform of heme-oxidized iron regulatory protein 2 [IRP2] ubiquitin ligase 1 [HOIL-1L] interacting protein (HOIP) and 2 accessory subunits called HOIL-1L and SHANK-associated RH domain-interacting protein (SHARPIN) (Figure 1A) (16). In addition to HOIP, which is the catalytic center for linear ubiquitination, HOIL-1L also contains a RING1-IBR-RING2 (RBR) ubiquitin ligase center (16). Previously, we showed that RBR on HOIL-1L attenuates LUBAC function by conjugating monoubiquitin onto all LUBAC subunits (20). Furthermore, we found that mice in which LUBAC activity is augmented by loss of the RING1 domain (HOIL-1L ΔRING1) exhibit immunological phenotypes, including activation of splenic B and T lymphocytes and elevated serum immunoglobulins, at a young age due to augmented LUBAC activity, which is itself caused by loss of HOIL-1L ligase activity (20).

Here, we found that HOIL-1L ∆RING1 mice spontaneously developed SLE and SS in a female-dominant manner. Furthermore, we found that a HOIL-1L variant, in which Arg464 is substituted for His (R464H) due to an rs774507518 missense single nucleotide polymorphism/variation (SNP/SNV) of the *RBCK1/HOIL-1L* gene, augmented LUBAC functions in human cells by attenuating HOIL-1L ligase activity. Importantly, the SNP/SNV was significantly enriched in patients with SLE and tended to be associated with primary SS, though this was not statistically significant due to limited statistical power. Collectively, the data suggest that HOIL-1L is the disease-associated gene product underlying SLE, and possibly SS, and that augmented LUBAC function underlies the pathogenesis of these autoimmune diseases.

## Results

### HOIL-1L ΔRING1 mice show SS-like symptoms.

We found that introduction of a HOIL-1L allele lacking ligase activity even at 1 locus augmented LUBAC function in mice by increasing linear ubiquitination of LUBAC substrates, leading to spontaneous activation of T and B lymphocytes and elevation of serum immunoglobulin levels at 10 weeks of age (Figure 1A) (20). Unexpectedly, we observed that HOIL-1L ∆RING1 mice older than 6 months exhibited opaque plaques on the cornea, with a female predominance (Figure 1, B and C, and Supplemental Figure 1A; supplemental material available online with this article; https://doi.org/10.1172/jci.insight.171108DS1). Keratinization of the cornea, a common feature of dry eye, was also observed (Figure 1D) (21, 22). H&E staining of lacrimal glands revealed extensive nodular infiltration of immune cells in not only 7-month-old HOIL-1L^∆RING1/∆RING1^ mice but also HOIL-1L^ΔRING1/+^ females, suggesting destruction of the gland via autoimmune mechanisms (Supplemental Figure 1B). Autoimmune-mediated dry eye is a characteristic symptom of SS, a systemic autoimmune disease observed predominantly in women (5). SS also involves autoimmune-mediated destruction of exocrine salivary glands (5, 22, 23). Indeed, we noted that the submandibular glands were also infiltrated by T and B lymphocytes and that some glands had B cell lymphoma 6–positive (BCL-6^+^) germinal centers (Figure 1, E and F). The titers of anti-Ro/SSA and anti-La/SSB autoantibodies, well-established biomarkers of SS (5), were higher in serum from HOIL-1L^ΔRING1/ΔRING1^ females than in that from wild-type (WT) mice (Figure 1, G and H). It is suggested that constitutive activation of NF-κB is related to the pathogenesis of SS (22–25). As shown in Figure 1I, expression of NF-κB target genes in the salivary glands of HOIL-1L^ΔRING1/ΔRING1^ female mice was markedly higher than that in WT mice, possibly due to augmented linear ubiquitination (Figure 1I). These data clearly indicate that female mice with augmented LUBAC activity induced by impairment of HOIL-1L E3 ligase activity spontaneously develop SS-like symptoms.

### HOIL-1L ΔRING1 mice develop lupus-like nephritis.

**Figure 2:**
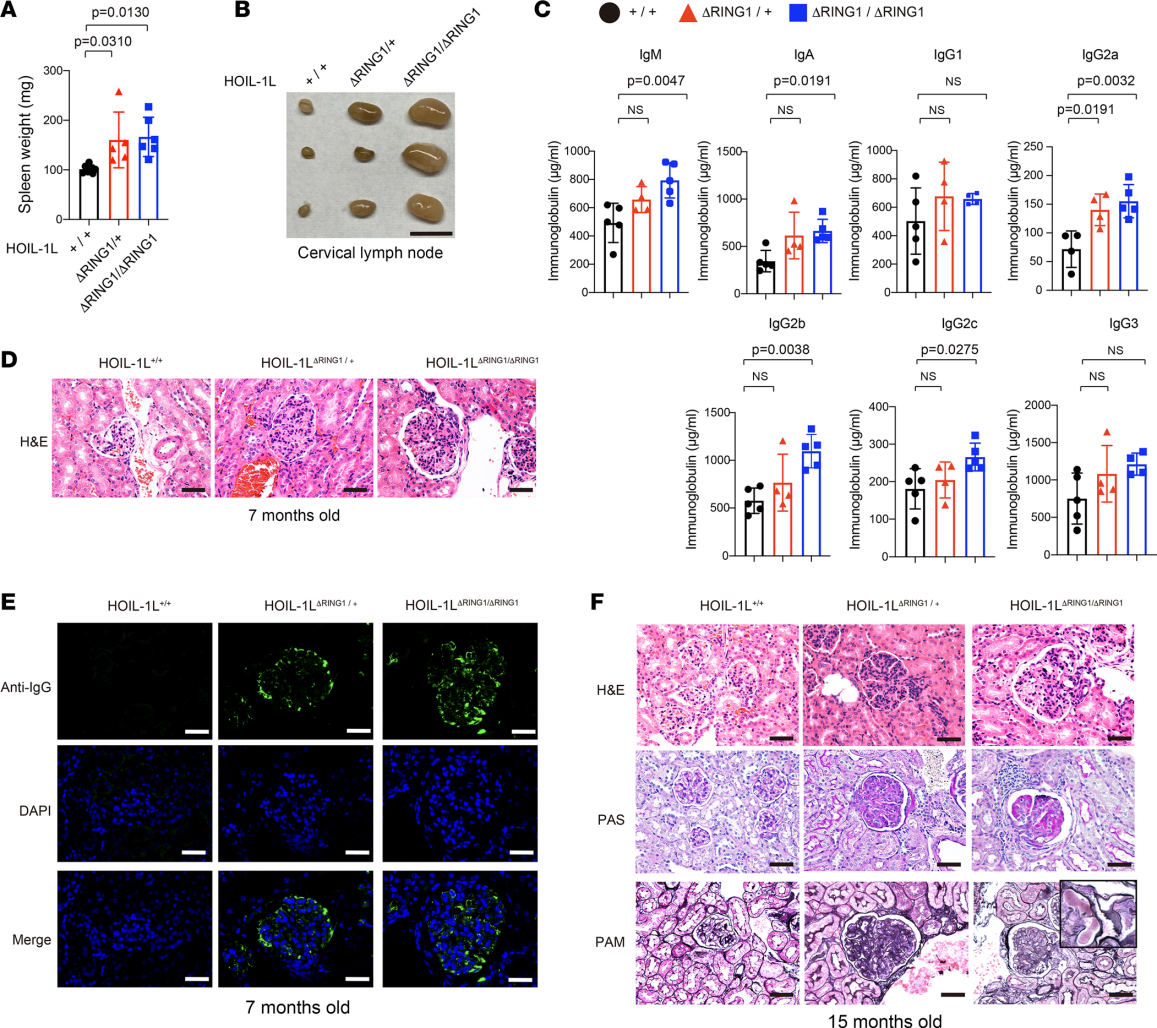
HOIL-1L ΔRING1 mice develop lupus-like nephritis. (**A**) Weight of spleens from 6- to 7-month-old female mice of the indicated genotype. Data are expressed as the mean ± SD. +/+ (*n* = 7), ΔRING1/+ (*n* = 5), and ΔRING1/ΔRING1 (*n* = 6). (**B**) Macroscopic picture of the cervical lymph nodes from the indicated littermate female mice at 7 months of age. Scale bars, 5 µm. *n* = 3 per genotype. (**C**) Titers of different immunoglobulin isotypes in the serum of littermate female mice (aged 6–7 months) of the indicated genotype were measured in an ELISA. Data are expressed as the mean ± SD. *n* = 4–5 per group. (**D**) Histological analysis was performed by H&E staining in the kidney from 7-month-old littermate female mice of the indicated genotype. Scale bars, 40 µm. (**E**) Histological analysis was performed by immunostaining sections of kidney from 7-month-old female littermates of the indicated genotype with an anti-IgG antibody and with DAPI. Scale bars, 40 µm. (**F**) Histological analysis, performed by H&E, periodic acid–Schiff (PAS), and periodic acid–Schiff-methenamine silver (PAM) staining, of kidney sections from 15-month-old littermate female mice of the indicated genotype. Scale bars, 40 µm. Inset, 4× magnification of background image. (**A** and **C**) *P* values were calculated by 1-way ANOVA, followed by Dunnett’s multiple comparisons test.

ABIN1 is a product of a disease susceptibility gene associated with SLE and SS (11, 25, 26). Recently, we reported that ABIN1 attenuates linear ubiquitin-mediated activation of NF-κB by degrading signaling protein complexes involved in the TLR signaling pathway (13), which implies that augmented linear ubiquitination is associated with SLE as well as SS. In addition to splenomegaly and lymphadenopathy, HOIL-1L ∆RING1 mice exhibited elevated serum immunoglobulins (Figure 2, A–C, and Supplemental Figure 2A), a phenomenon often observed in patients with SLE. Therefore, we asked whether HOIL-1L ∆RING1 mice exhibit lupus-like symptoms, including lupus nephritis (1, 2, 27). Histopathologic assessment revealed proliferation of mesangial cells, with deposition of IgG and complement components C3 and C1q (all characteristic features of lupus nephritis) in the glomerulus of 7-month-old HOIL-1L^ΔRING1/ΔRING1^ female mice (Figure 2, D and E, and Supplemental Figure 2, B and C). Furthermore, deposition of immune complexes and mesangial proliferation were observed not only in homozygous HOIL-1L ∆RING1 mice but also in HOIL-1L^ΔRING1/+^ female mice, albeit to a lesser extent in the latter (Figure 2, D and E, and Supplemental Figure 2, B and C). It is of note that the lupus nephritis–like symptoms appeared more severe in female than in male mice, which reflects the female predominance of SLE in humans (Supplemental Figure 2, D and E). Enlarged glomeruli with a PAS-positive glomerular basement membrane were detected in 15-month-old female mice lacking HOIL-1L ligase at least 1 locus, which implies that nephritis progresses with age, albeit slowly (Figure 2F). These data suggest that augmented linear ubiquitination is involved in the pathogenesis of lupus nephritis.

### HOIL-1L ΔRING1 mice show most characteristic features of lupus.

**Figure 3:**
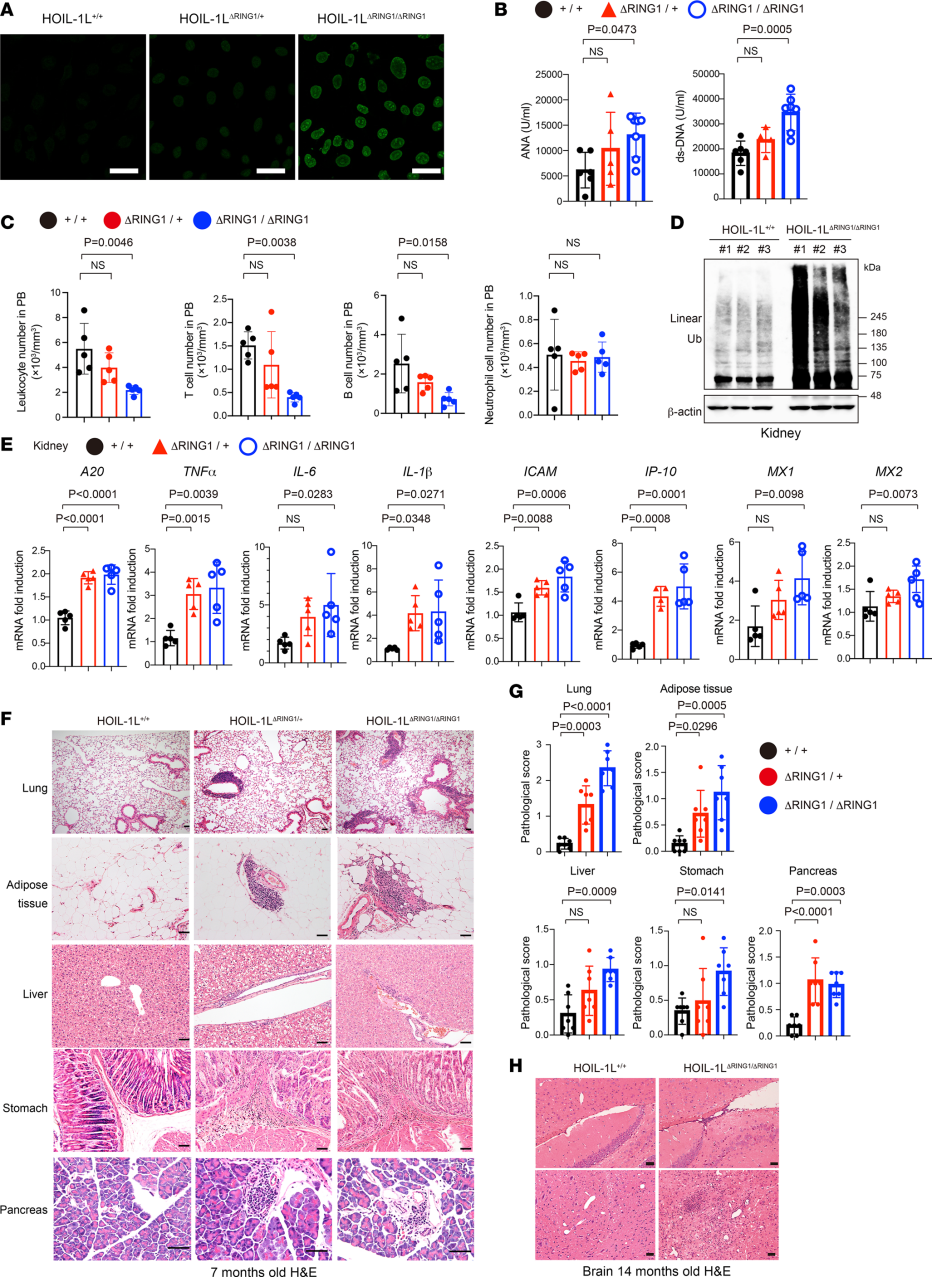
HOIL-1L ΔRING1 mice show most characteristic features of lupus. (**A**) Hep-2 immunofluorescence analysis of ANA in 7-month-old female mice of the indicated genotype. Scale bars, 40 µm. (**B**) Levels of anti-ANA and anti-dsDNA antibodies in the serum from 7- to 10-month-old female mice of the indicated genotype. *n* = 4–7 per group. (**C**) Number of leukocytes, T cells, B cells, and neutrophils in the peripheral blood of female mice of the indicated genotype (aged 7 months; *n* = 5 per genotype). (**D**) Lysates of kidneys from littermate female mice of the indicated genotypes (aged 3–4 months) were subjected to immunoblotting to detect linear ubiquitin chains. *n* = 3 per genotype. (**E**) QPCR analysis of kidneys from 3- to 4-month-old female mice of the indicated genotype (*n* = 5 per genotype). (**F**) H&E staining of multiple organs from 7-month-old female littermate mice of the indicated genotype. Scale bars, 50 µm. (**G**) Quantification of cellular infiltration into the organs of 7-month-old female mice of the indicated genotype (*n* = 7 per genotype). (**H**) H&E staining of brain sections from littermate female mice (14 months old) of the indicated genotype. Scale bars, 50 µm. (**B**, **C**, **E**, and **G**) Data are expressed as the mean ± SD. *P* values were calculated by 1-way ANOVA, followed by Dunnett’s multiple comparisons test.

In addition to anti-nuclear antibodies (ANA), we detected anti-dsDNA antibodies, an autoantibody characteristic of lupus nephritis (1–3), in the serum of both HOIL-1L^ΔRING1/ΔRING1^ and HOIL-1L^ΔRING1/+^ mice, and the titer appeared to be related to the number of HOIL-1L ∆RING1 alleles (Figure 3, A and B). Hematologic examination revealed significant lymphocytopenia, which is often observed in patients with SLE; this was particularly prominent in HOIL-1L^ΔRING1/ΔRING1^ mice, though neutrophil counts and the T/B and CD4^+^/CD8^+^ T lymphocyte ratios were the same (Figure 3C and Supplemental Figure 3, A–C). HOIL-1L^ΔRING1/+^ mice also demonstrated a trend toward low lymphocyte counts, despite this not reaching statistical significance (Figure 3C). Furthermore, before developing nephritis, the amount of linear ubiquitin increased markedly, as did transcripts of NF-κB target genes such as A20 and TNF-α, in the kidneys of HOIL-1L^ΔRING1/ΔRING1^ and HOIL-1L^ΔRING1/+^ female mice from as early as 3 months of age (Figure 3, D and E), suggesting that these phenomena underlie the pathogenesis of nephritis. The amount of linear ubiquitin detected in multiple organs (including the kidney) of HOIL-1L^ΔRING1/+^ mice increased (Supplemental Figure 3D). Furthermore, increased expression of type I IFN-stimulated genes (ISGs), known as the “IFN signature,” is commonly observed in patients with SLE (1–3, 28, 29). It is of note that in addition to NF-κB, transcription of type I ISGs *IP-10*, *MX1*, *MX2*, and *IRF5* was upregulated in the kidneys of mice harboring 1 or 2 HOIL-1L ∆RING1 loci (Figure 3E and Supplemental Figure 3E).

We also found that organs other than the kidney were damaged in mutant mice, again resembling findings in patients with SLE (1, 2). The lung, adipose tissue, liver, stomach, and pancreas of HOIL-1L ∆RING1 mice were mildly infiltrated by immune cells at young stage (2 months old), and these infiltrations were getting worse with age at adult stage (7 months old) (Figure 3, F and G, and Supplemental Figure 3F). Infiltration of perivascular lesions and the brain parenchyma, mainly B and T lymphocytes, was observed in HOIL-1L ∆RING1 mice, only at old age (more than about 12 months old) (Figure 3H and Supplemental Figure 3G). No infiltration of the joints and skins was observed even at old age (Supplemental Figure 3, H and I). These results clearly indicate that introduction of even 1 HOIL-1L ∆RING1 allele is sufficient to cause SLE-like symptoms in mice via augmented linear ubiquitination activity.

### Activation of both B and T cells in HOIL-1L ΔRING1 mice.

**Figure 4:**
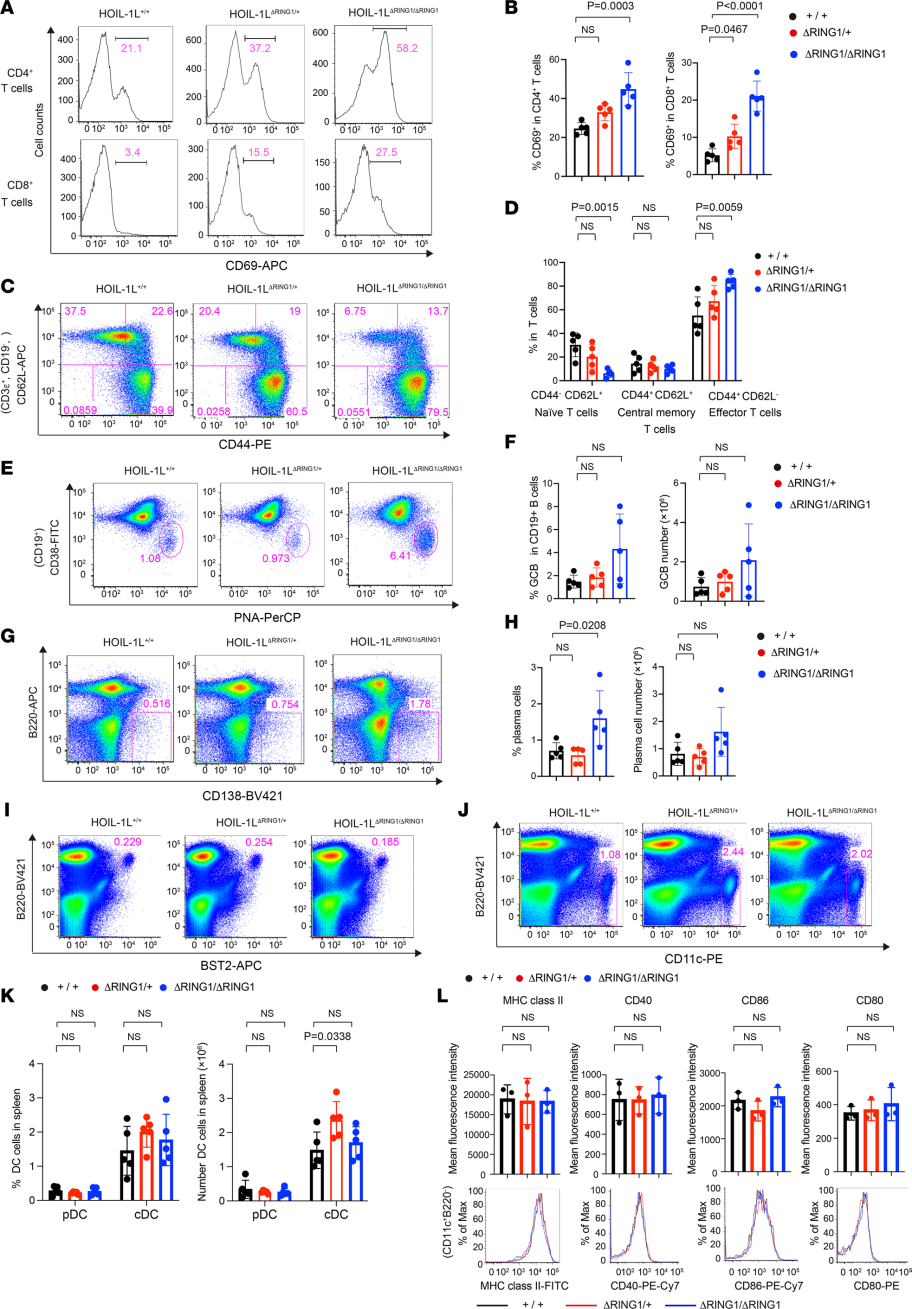
Activation of both B and T cells in HOIL-1L ΔRING1 mice. (**A**–**L**) Representative flow cytometry plots and quantification of splenocytes from 7- to 8-month-old female mice of the indicated genotypes. *n* = 5 per genotype. (**A** and **B**) Surface expression of CD69 by CD4^+^ and CD8^+^ T cells. (**C** and **D**) CD3ε^+^CD19^–^ T cells were analyzed for surface expression of CD44 and CD62L. Naive T cells (CD44^–^CD62L^+^), central memory T cells (CD44^+^CD62L^+^), and effector T cells (CD44^+^CD62L^–^). (**E** and **F**) CD19^+^ B cells were analyzed for surface expression of CD38 and peanut agglutinin (PNA). (**F**) Percentage and number of GCB cells (PNA^+^CD38^lo^). (**G** and **H**) Surface expression of B220 and CD138. (**H**) Percentage and number of plasma cells (B220^lo^CD138^hi^). (**I**–**K**) Plasmacytoid dendritic cells (pDC), B220^+^BST2^+^; conventional dendritic cells (cDC), CD11c^+^B220^–^. (**L**) CD11c^+^B220^–^ cDCs were analyzed for surface expression of MHC class II, CD40, CD86, and CD80. *n* = 3 per genotype. (**B**, **D**, **F**, **H**, **K**, and **L**) Data are expressed as the mean ± SD. *P* values were calculated by 1-way ANOVA, followed by Dunnett’s multiple comparisons test.

Next, we examined immunological changes in the spleen. Although neither the total number of splenocytes nor the T/B lymphocyte ratio differed between WT and HOIL-1L ∆RING1 mice, the CD4^+^/CD8^+^ T cell ratio increased slightly in the latter (Supplemental Figure 4, A–E). Expression of CD69, an activation marker of T cells, by CD4^+^ and CD8^+^ T cells from HOIL-1L^ΔRING1/+^ and HOIL-1L^ΔRING1/ΔRING1^ mice increased significantly (Figure 4, A and B). More CD44^+^CD62L^–^ effector T cells, and fewer CD44^–^CD62L^+^ naive T cells, were observed in HOIL-1L^ΔRING1/+^ and HOIL-1L^ΔRING1/ΔRING1^ mice (Figure 4, C and D), implying that T cells are constitutively activated and are in a proinflammatory state under conditions of augmented LUBAC ligase activity. The proportion of germinal center B (GCB) cells and plasma cells in HOIL-1L ∆RING1 mice was higher than in WT mice, although the difference was not significant (Figure 4, E–H, and Supplemental Figure 4F), which appears compatible with the hypergammaglobulinemia observed in HOIL-1L ∆RING1 mice (Figure 2C) (20). Furthermore, the proportion of marginal zone, but not follicular, B cells tended to be higher in HOIL-1L ∆RING1 mice (Supplemental Figure 4, G and H). There was no significant difference in the proportion or number of plasmacytoid dendritic cells (pDCs), conventional dendritic cells (cDCs), and neutrophils (Figure 4, I–K, and Supplemental Figure 4I). There was no clear difference in expression of activation markers for cDCs (i.e., MHC class II, CD40, CD86, and CD80) between HOIL-1L^+/+^ and HOIL-1L^ΔRING1/ΔRING1^ mice (Figure 4L). Furthermore, a dihydrorhodamine (DHR) 123 flow cytometry assay to detect production of reactive oxygen species (ROS) upon treatment of neutrophils with phorbol 12-myristate 13-acetate (PMA) revealed no significant difference between HOIL-1L^+/+^ and HOIL-1L^ΔRING1/ΔRING1^ mice (Supplemental Figure 4, J and K) (30, 31). Collectively, these results clearly demonstrate global activation of splenic lymphocytes in HOIL-1L ∆RING1 mice under steady-state conditions, even in those with only 1 locus of HOIL-1L ∆RING1.

### Augmented TLR signaling in HOIL-1L ΔRING1 mice.

**Figure 5:**
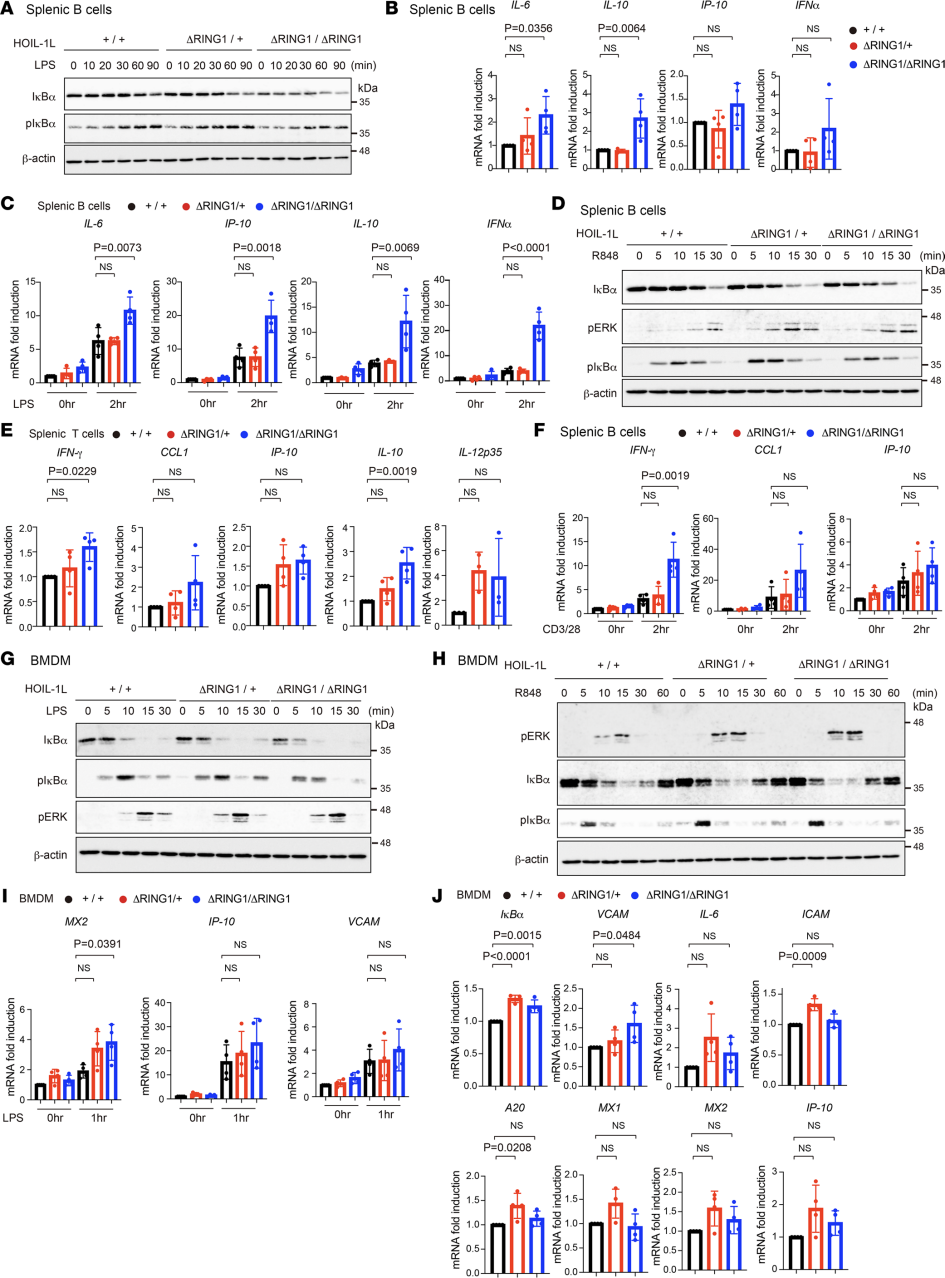
Augmented TLR signaling in HOIL-1L ΔRING1 mice. (**A** and **D**) Splenic primary B cells from 12-week-old littermate female mice of the indicated genotype were treated with LPS (20 mg/mL) (**A**) or R848 (1 mg/mL) (**D**) for the indicated times, then assessed by immunoblotting with the indicated antibodies. (**B** and **C**) QPCR analysis of splenic primary B cells from 10- to 12-week-old female mice of the indicated genotype. Splenic B lymphocytes were treated with or without LPS (20 mg/mL) for the indicated times, followed by qPCR (**C**). *n* = 4 per genotype. For each target, data sets of **B** and **C** (0 hours) are the same. (**E** and **F**) QPCR analysis of splenic T cells from 10- to 12-week-old female mice of the indicated genotype. Splenic primary T cells were treated with CD3 (1 mg/mL) and CD28 (1 mg/mL) for the indicated times, followed by qPCR (**F**). *n* = 4 per genotype. For each target, data sets of **F** (0 hours) and **E** are the same. (**G** and **H**) BMDMs from 12-week-old littermate female mice of the indicated genotype were treated with LPS (10 mg/mL) (**G**) or R848 (1 mg/mL) (**H**) for the indicated times and assessed by immunoblotting with the indicated antibodies. (**I** and **J**) QPCR analysis from BMDMs from 10- to 12-week-old female mice of the indicated genotype. BMDMs were treated with LPS (10 ng /mL) for the indicated times, followed by qPCR (**I**). *n* = 4 per genotype. For each target, data sets of **I** (0 hours) and **J** are the same. (**B**, **C**, **E**, **F**, **I**, and **J**) Data are expressed as the mean ± SD. *P* values were calculated by 1-way ANOVA, followed by Dunnett’s multiple comparisons test.

Because B lymphocytes were activated in HOIL-1L ∆RING1 mice, we evaluated the responses of splenic B cells to TLR ligands (Supplemental Figure 5, A–C). Degradation of IκBα, a hallmark of activation of NF-κB signaling (32), and expression of NF-κB target genes induced by LPS, a TLR4 ligand, were enhanced in HOIL-1L ΔRING1 mice. Augmented LUBAC activity also increased LPS-induced expression of type I ISGs, as well as IFN-α, in addition to NF-κB (Figure 5, A–C). Stimulation of both TLR7 and TLR9 by R848 and CpG-DNA, respectively, also augmented NF-κB signaling in HOIL-1L ∆RING1 mice (Figure 5D and Supplemental Figure 5D). Supporting the data regarding increased numbers of effector T lymphocytes (Figure 4, C and D), splenic T cells from HOIL-1L ∆RING1 mice expressed cytokines, such as IFN-γ, in the absence of any stimulation (Figure 5E and Supplemental Figure 5E); CD3/CD28 stimulation further augmented production of cytokine mRNAs (Figure 5F). Since LUBAC-mediated linear ubiquitination is essential for immune signaling in not only B and T lymphocytes but also myeloid cells (33–35), we stimulated bone marrow–derived macrophages (BMDMs) with LPS (Supplemental Figure 5, F and G). Increased phosphorylation and degradation of IκBα and phosphorylation of ERK were observed in LPS-stimulated BMDMs derived from HOIL-1L ∆RING1 mice (Figure 5G), suggesting that not only NF-κB but also ERK signaling was augmented by enhanced LUBAC functions via loss of HOIL-1L ligase activity (20, 33). Furthermore, stimulation of TLR7 by R848 augmented not only NF-κB but also ERK signaling in HOIL-1L ∆RING1 mice (Figure 5H). LPS-induced expression of type I IFN-inducible genes was augmented in HOIL-1L ∆RING1 BMDMs (Figure 5I and Supplemental Figure 5H). It is noteworthy that augmented LUBAC activity tended to increase expression of not only NF-κB target genes but also type I IFN and its inducible genes in BMDMs derived from HOIL-1L ∆RING1 mice in the absence of stimulation (Figure 5J and Supplemental Figure 5I). Collectively, these results clearly suggest that augmented LUBAC activity hyperactivates BMDMs as well as T and B cells.

### The HOIL-1L R464H variant shows reduced E3 activity to trigger formation of LUBAC-mediated linear ubiquitin chains.

Our analyses of HOIL-1L ∆RING1 mice showed that impaired ligase activity, even at 1 allele of HOIL-1L, underlies lupus and SS. Since we showed that a Cys 460 to Ala substitution (C460A) abolishes the E3 activity of HOIL-1L (20), we suspected that missense SNPs/SNVs of HOIL-1L might play a role in human SLE and SS by attenuating its ligase activity. Therefore, we looked for missense variants in the catalytic RBR center of HOIL-1L, which is encoded by the *RBCK1* gene, using the public Genome Aggregation Database (gnomAD) (36) and an in-house whole-genome sequencing (WGS) data set comprising 3,026 high-depth Japanese WGS samples (JEWEL 3K) (7). We found 31 SNVs in HOIL-1L RBR (Supplemental Figure 6A). Among these, we looked for SNVs that augmented LUBAC functions via attenuation of HOIL-1L E3 ligase activity.

To do this, we introduced HOIL-1L variants harboring the 31 SNVs into HEK293T cells, together with the other 2 LUBAC components (HOIP and SHARPIN). We found that 8 of the 31 SNVs encoded HOIL-1L variants that reproducibly augmented generation of linear ubiquitin chains when compared with the WT (Supplemental Figure 6B). The luciferase reporter analyses revealed that among the 8 HOIL-1L variants, 2, Leu435 to Pro (L435P) (registered as SNP/SNV rs1208529314) and Arg464 to His (R464H) (registered as SNP/SNV rs774507518), appeared to increase LUBAC-mediated NF-κB activation (Supplemental Figure 6C). Next, we asked whether the 2 SNVs encoding the 2 HOIL-1L variants were enriched in human patients with SLE. We extracted GWAS results for 148,312 Japanese samples (2,535 SLE cases versus 145,777 controls) imputed with a reference panel built from JEWEL 3K and the 1000 Genome Project (1KGP) data set. While rs1208529314 was not found in the data set, rs774507518, encoding HOIL-1L R464H, showed significant enrichment in SLE (OR = 3.87, *P* = 8.2 **×** 10^–6^) (Table 1).

To explore the potential association between SNP rs774507518 and SS, we used microarray data from 626 Japanese cases (378 primary SS and 248 secondary SS, coexisting with other rheumatic diseases) genotyped by the Illumina Infinium Omni Express Exome array, along with 140,248 Japanese controls from the BioBank Japan project (BBJ) (7). After standard quality control of the microarray data, we performed local imputation of the flanking region of *RBCK1/HOIL-1L* (chr20:1–5,000,000) with the same reference panel used for SLE analysis and conducted logistic regression analysis. We found that the *RBCK1/HOIL-1L* SNV encoding R464H (rs774507518) showed a trend toward an association with primary SS (OR: 2.10, *P* = 0.19, Table 2), though the result was not significant because of limited statistical power.

**Figure 6:**
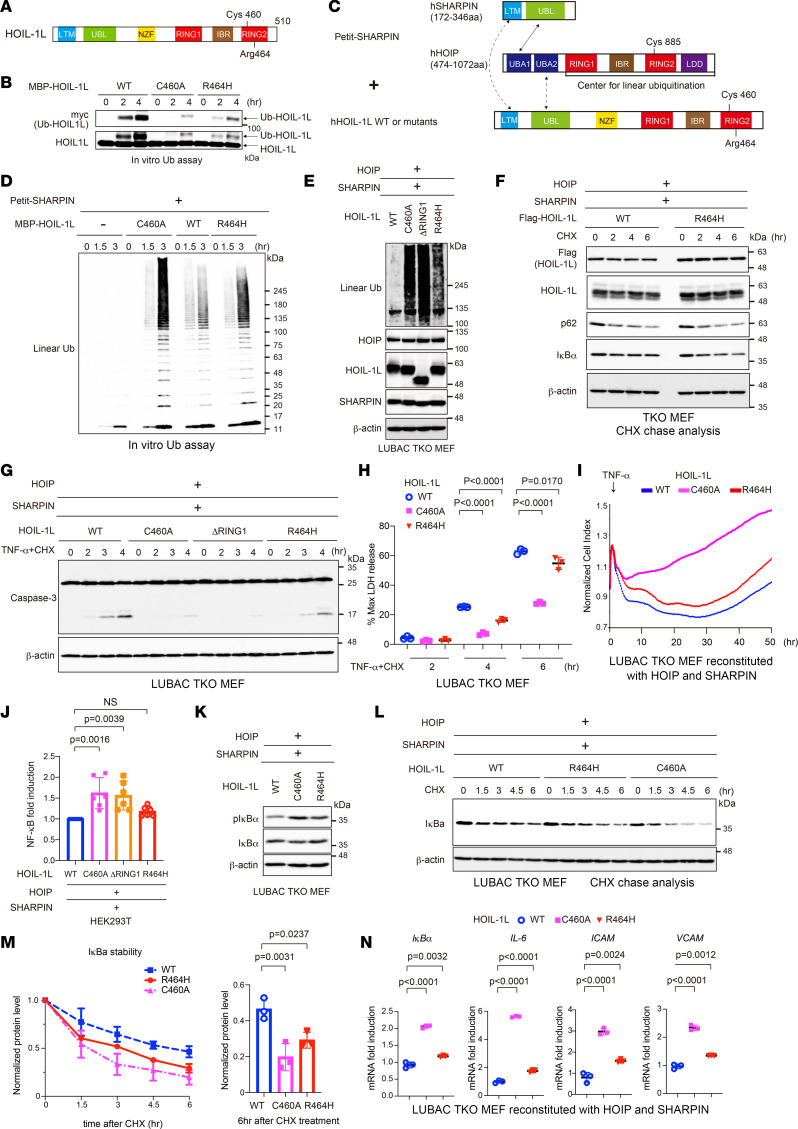
The HOIL-1L R464H variant shows reduced E3 activity to trigger formation of LUBAC-mediated linear ubiquitin chains. (**A** and **C**) Schematic representation of HOIL-1L and the Petit-SHARPIN domains and the experimental protocols for **D**. (**B**) Auto-monoubiquitination of HOIL-1L in the presence of the indicated recombinant HOIL-1L and Myc-tagged ubiquitin. (**D**) Generation of linear ubiquitin chains in the presence of the indicated recombinant proteins in in vitro ubiquitination assays. (**E** and **K**) Lysates from LUBAC-TKO MEFs stably reconstituted with the indicated proteins were probed as indicated. (**F**) Stability of HOIL-1L, p62, and IκBα in LUBAC-TKO MEFs expressing the indicated proteins and treated with CHX (20 mg/mL). (**G**) Lysates from LUBAC-TKO MEFs stably reconstituted with the indicated proteins, then stimulated with TNF-α (1 ng/mL) and CHX (20 mg/mL), were probed as indicated. (**H**) Cell death of LUBAC-TKO MEFs stably reconstituted with HOIP, SHARPIN, and HOIL-1L, followed by TNF-α (10 ng /mL) and CHX (20 mg/mL), was monitored by measuring lactate dehydrogenase (LDH) activity. Mean values (*n* = 3) are shown. (**I**) Viability of LUBAC-TKO MEFs stably reconstituted with HOIP, SHARPIN, and HOIL-1L (as indicated), then stimulated with TNF-α (40 ng/mL), was measured using the iCELLigence. (**J**) NF-κB activation in HEK293T cells transfected with the indicated expression plasmids was measured in a luciferase assay. Mean values (*n* = 6) ± SD are shown. (**L** and **M**) Stability of IκBα in LUBAC-TKO MEFs expressing the indicated proteins and treated with CHX (20 mg/mL). Data are expressed as the mean (*n* = 3) ± SD. (**N**) QPCR analysis of TKO MEFs stably reconstituted with the indicated proteins. Mean values (*n* = 3) ± SD are shown. (**H**, **J**, **M**, and **N**) *P* values were calculated by Dunnett’s multiple comparisons test.

We then tried to verify whether HOIL-1L R464H augments LUBAC ligase functions by attenuating the ligase activity of HOIL-1L. HOIL-1L monoubiquitinates LUBAC subunits, including HOIL-1L itself (auto-monoubiquitination), to suppress LUBAC function (20). An in vitro ubiquitination assay revealed that the R464H variant attenuated the monoubiquitination activity of HOIL-1L though less effectively than the ligase-null mutant HOIL-1L C460A (Figure 6, A and B). To examine whether HOIL-1L R464H augmented linear ubiquitination by HOIP, HOIL-1L variants were subjected to an in vitro ubiquitination assay together with Petit-SHARPIN, which comprises fragments of HOIP and SHARPIN (20). HOIL-1L R464H potentiated the linear ubiquitination activity of Petit-SHARPIN to a greater extent than HOIL-1L WT (Figure 6, C and D). Furthermore, introduction of HOIL-1L R464H plus HOIP and SHARPIN led to a marked increase in the number of linear ubiquitin chains in HEK293T cells (Supplemental Figure 6D). Since amino acid substitutions can affect protein stability, we evaluated the effect of the R464H substitution on the stability of HOIL-1L. When HOIL-1L, along with HOIP and SHARPIN, were introduced into mouse embryonic fibroblasts (MEFs) lacking all LUBAC subunits (triple-knockout [TKO] MEFs) using a retrovirus transduction system (20, 37), the amount of HOIL-1L R464H was comparable with that of HOIL-1L WT in reconstituted TKO MEFs, and HOIL-1L R464H generated more linear ubiquitin chains than HOIL-1L WT (Figure 6E). Cycloheximide (CHX) decay analysis revealed that HOIL-1L R464H appeared to be as stable as the WT (Figure 6F and Supplemental Figure 6E). Since HOIL-1L R464H was stable in cells and augmented the linear ubiquitination activity of LUBAC, we evaluated whether HOIL-1L R464H indeed enhanced LUBAC functions (i.e., NF-κB activation and protection from cell death). HOIL-1L R464H suppressed TNF-α–mediated activation of caspase-3, a hallmark of apoptosis, more effectively than the WT, though less effectively than ligase-defective HOIL-1L C460A (Figure 6, G–I). The NF-κB reporter assay revealed that HOIL-1L R464H appeared to activate NF-κB more potently than the WT (Figure 6J and Supplemental Figure 6C). In the absence of stimulation, phosphorylation of IκBα was augmented, and the CHX chase analyses verified increased turnover of IκBα (Figure 6, K–M). Transcription of NF-κB target genes was also activated in cells expressing HOIL-1L R464H (Figure 6N). Furthermore, to investigate the effects of the HOIL-1L R464H variant in human immune cells, HOIL-1L WT or mutant HOIL-1L, along with HOIP and SHARPIN, were introduced into a human leukemia monocytic cell line, THP-1, using a retrovirus transduction system. Augmented production of linear ubiquitin chains was observed in cells expressing HOIL-1L R464H in the absence of stimulation, validating enhanced LUBAC activity via impairment of HOIL-1L ligase activity (Supplemental Figure 6F). Transcription of NF-κB target genes, and phosphorylation and degradation of IκBα, were also augmented in LPS-stimulated THP-1 cells expressing HOIL-1L R464H (Supplemental Figure 6, G and H), which clearly validated that HOIL-1L R464H increases NF-κB activation by augmenting LUBAC functions.

Collectively, these results strongly indicate that the HOIL-1L R464H variant significantly enhances LUBAC functions by attenuating the ligase activity of HOIL-1L, resulting in susceptibility to SLE.

## Discussion

In this study, we demonstrate that attenuated HOIL-1L ligase activity causes lupus and SS in mice by augmenting LUBAC-mediated linear ubiquitination. We also sought to identify *RBCK1*/*HOIL-1L* SNPs/SNVs that attenuate HOIL-1L ligase activity and found that the *RBCK1/HOIL-1L* SNV (rs774507518) encoding HOIL-1L R464H showed significant enrichment in SLE (Table 1). To the best of our knowledge, this study is the first to show that HOIL-1L, a component of LUBAC, is the disease susceptibility gene product for SLE. Although more than 150 susceptibility loci are associated with SLE, most have been identified in GWAS analyzing genetic variations commonly observed in a population (~0.5%); these studies reported weak associations with SLE (1–3). Here, we identified *RBCK1/HOIL-1L* as an SLE susceptibility gene in mice and obtained human genetic evidence based on a very rare variant showing a strong association with SLE to support the murine findings.

*HOIL-1L*/*RBCK1* has been identified as a gene that causes autoinflammation with immunodeficiency and/or polyglucosan storage myopathy type 1 (PGBM1) (38–42). Both alleles of *RBCK1* are mutated in these patients, and the majority of identified mutations are truncated or frameshift mutations, suggesting that loss of intact HOIL-1L underlies these diseases (38–40). HOIL-1L plays 2 totally conflicting roles in LUBAC function: stabilization of the LUBAC complex and suppression of LUBAC function (20, 37). HOIL-1L stabilizes LUBAC by interacting with the HOIP and SHARPIN subunits through the UBL and LTM domains, respectively, located at the N-terminus of HOIL-1L (Figure 1A) (37). It is proposed that the positions of mutations in HOIL-1L are related to the symptoms observed in patients. Mutations causing loss of expression or loss of function of the N-terminal regions of HOIL-1L have been identified in patients with autoinflammation and immunodeficiency (39). By contrast, mutations in the middle or C-terminal regions of HOIL-1L have been reported in PGBM1 without immunodeficiency (38–41). It is hypothesized that mutations in the N-terminal region of HOIL-1L reduce the amount of LUBAC more profoundly than those in the mid- or C-terminal regions; this is because the N-terminal region plays crucial roles in complex formation by interacting with other LUBAC subunits (37).

Here, we found that the HOIL-1L variant causing SLE is defective in another function of HOIL-1L: inhibition of LUBAC-mediated linear ubiquitination via C-terminal RBR ligase domain of HOIL-1L (20, 37, 43). To augment LUBAC functions, the variant must exhibit 2 criteria: suppression of the ligase activity of HOIL-1L and no or only a minor effect on HOIL-1L stability. Some HOIL-1L mutations that cause PGBM1 lead to loss of the ligase center from HOIL-1L (38, 39). Although 1 allele of HOIL-1L having attenuated ligase activity is sufficient to induce SLE, no reports show that parents of patients with PGBM1 harboring the mutation in 1 allele of HOIL-1L exhibit any overt symptoms. This may be because the mutations in PGBM1 reduce the amount of HOIL-1L protein, which reduces the chance of developing the disease. Next, we examined the reported *RBCK1/HOIL-1L* SNPs/SNVs in the context of the 2 criteria and found that among the reported *RBCK1* SNPs/SNVs in the RBR region, only 2 (rs1208529314 and rs774507518) appeared to be suitable candidates as causative agents of SLE (Supplemental Figure 6A). We then identified SNV rs774507518, encoding HOIL-1L R464H, as an SLE susceptibility variant. It is not very surprising that rs774507518 is very rare considering its biological impact on HOIL-1L. Therefore, we suspect that it is almost impossible to identify RBCK1/HOIL-1L as an SLE susceptibility gene using conventional GWAS approaches.

Female mice harboring augmented LUBAC activity develop lupus and SS symptoms. Despite the fact that the molecular mechanism underlying the striking female predominance of SLE and SS remains unidentified (1, 44), recent studies suggest involvement of the X chromosome gene dose. Although dosage compensation for X-linked gene products between XX females and XY males is achieved by X chromosome inactivation (XCI) during the early development of female embryos, around 20% of X-linked genes show incomplete XCI, and several escape from XCI, resulting in overdosage of the gene products (45). However, this is not the case with *RBCK1/HOIL-1L* because the *RBCK1* gene resides on chromosomes 2 and 20 in mice and humans, respectively (38). Several genes encoding proteins involved in immune signaling evade XCI (46). It is of great interest that *TLR7* escapes XCI in some cells, including B lymphocytes and myeloid cells (45), because TLR7 plays a key role in the pathogenesis of SLE (47). An increased copy number of *TLR7* causes SLE-like symptoms, and deletion of *TLR7* attenuates symptoms, in lupus-prone mice (18). TLR7 induces production of IFN-α and activates NF-κB via myeloid differentiation factor 88–dependent (MyD88-dependent) signaling. LUBAC-mediated linear ubiquitination plays a mandatory role in TLR/MyD88 signaling because, as we clearly showed, loss of HOIL-1L ligase activity augments NF-κB activation induced not only by LPS and CpG-DNA (ligand of TLR9) but also by R848 (ligand of TLR7), in splenic B cells and BMDMs (Figure 5, A, C, D, and G–I, and Supplemental Figure 5, D and H). It is noteworthy, however, that no overt female predominance of SLE-like symptoms was reported in mice expressing the ABIN1 mutant lacking linear ubiquitin-binding activity (17, 19), although loss of linear binding of ABIN1 augments LUBAC functions, as is the case with loss of HOIL-1L ligase activity (17). We showed that male HOIL-1L ΔRING1 mice also exhibit lupus-like symptoms, albeit mild. Since the symptoms observed in ABIN1-mutant mice are more severe than those in HOIL-1L ΔRING1 mice (19), it seems likely that female dominance was not observed in ABIN1-mutant mice due to disease severity. Therefore, we suspect that augmented TLR signaling underlies exacerbated lupus-like symptoms in female HOIL-1L ΔRING1 mice.

Although dermatitis is a common feature of patients with SLE, HOIL-1L ∆RING1 mice do not exhibit dermatitis, as is the case with most murine lupus models (except MRL/lpr mice) (48) and ABIN1 D485N mice (18). Although the exact reason underlying the lack of skin lesions in most lupus model mice remains unknown, it might be due to biological differences between mice and humans. Alternatively, environmental factors may play a role. Indeed, ultraviolet (UV) rays and infection by some viruses can contribute to lupus dermatitis; model mice are not exposed to UV or infectious pathogens because they are housed in a specific pathogen–free environment.

In addition to HOIL-1L, 3 well-established SLE susceptibility genes are associated with augmented linear ubiquitin signaling (1, 2, 9, 26, 27, 49–53). The first is *TNIP1/ABIN1* (27, 51). SLE susceptibility SNPs in *TNIP1/ABIN1* augment linear ubiquitination by reducing expression of ABIN1 (9, 26, 27, 50, 51); this is because ABIN1 suppresses LUBAC functions acting as a linear chain-specific autophagy adaptor (13). Another gene is *UBE2L3* (9, 49, 50, 52, 53). Ubiquitin is conjugated to substrates via a cascade reaction mediated by 3 kinds of enzymes: ubiquitin activating enzyme (E1), ubiquitin conjugating enzyme (E2), and ubiquitin ligase (E3) (54, 55). The LUBAC ligase complex is an E3 enzyme. The E2 that functions coordinately with LUBAC during linear ubiquitination is UBE2L3 (49, 56). The SLE risk allele of *UBE2L3* increases expression of UBE2L3 protein, which then enhances LUBAC-mediated activation of NF-κB (49). The third gene is *TNFAIP3* (8, 57). TNFAIP3 suppresses NF-κB activation by binding to linear ubiquitin chains via its zinc finger 7 domain (58, 59), and the SLE susceptibility SNPs of *TNPAIP3* induce hypomorphic expression of TNFAIP3, which appears to augment linear ubiquitination signaling. Also, SLE susceptibility SNPs of *TNFAIP3* interact with those of *UBE2L3*, and these variations act synergistically to activate NF-κB, thereby increasing the risk of lupus (60). Collectively, our data show that LUBAC-mediated linear ubiquitin signaling is very closely associated with the pathogenesis of SLE. Since LUBAC-mediated linear ubiquitination augments both innate and acquired immunity, it is of great interest to examine whether inhibiting LUBAC function or suppressing linear ubiquitin signaling will be a novel therapeutic approach for SLE.

## Methods

### Mice.

HOIL-1L ∆RING1 mice were generated previously (20). All animal studies were approved by the Animal Research Committee, Graduate School of Medicine, Kyoto University, and all experiments complied with all relevant ethical regulations regarding animal research.

### Histology and immunohistochemistry.

Samples were fixed in 4% buffered paraformaldehyde, followed by embedding in paraffin. Most organs were stained with H&E, and some paraffin-embedded specimens of kidneys were stained with PAS and PAM. For immunohistochemistry, after antigen retrieval for 10 minutes at 99°C in citric buffer (pH 6.0), slides were blocked for 30 minutes with PBS containing 2% BSA, 5% goat serum, and 0.1% Triton X-100; incubated with a primary antibody for 16 hours at 4°C; and incubated for 1 hour at room temperature with the appropriate Alexa Fluor 488–conjugated secondary antibody (1:200 dilution). After washing, the samples were mounted with a coverslip using ProLong Gold antifade reagent containing DAPI (Thermo Fisher Scientific) and then imaged on a BZ-900 fluorescence microscope (Keyence). For peroxidase staining, slides were treated with 3% H_2_O_2_ for 10 minutes at room temperature, washed, blocked, and incubated with a primary antibody. After washing, the slides were incubated for 20 minutes with Simple Stain Mouse MAX-PO (Nichirei Biosciences), washed, incubated with DAB (Pierce), and immediately washed and counterstained with hematoxylin prior to imaging on a BX51 microscope (Olympus). The following primary antibodies were used for immunohistochemistry: anti-keratin14 (PRB-155P, BioLegend), anti-CD3ε (sc-1127, Santa Cruz Biotechnology), anti-CD45R (B220) (103202, BioLegend), anti-C3 (ab200999, Abcam), anti-C1q (JL-1, Hycult), anti-mouse IgG FITC (F2266, MilliporeSigma), and anti–BCL-6 (sc-7388, Santa Cruz Biotechnology). The following secondary antibodies were used for immunohistochemistry: goat anti-mouse IgG (A-11029, Life Technologies), goat anti-rabbit IgG (A-11034, Life Technologies), donkey anti-goat IgG (A-11055, Molecular Probes), and goat anti-rat IgG (112–545-167, Jackson ImmunoResearch).

### Real-time quantitative reverse transcription PCR.

Tissue samples from kidneys and submandibular glands were minced into small pieces on ice using scissors and then homogenized in a tissue homogenizer (TissueRuptor, QIAGEN). RNA was extracted with ISOGEN (Nippongene), according to the manufacturer’s protocol. RNAs were extracted from BMDMs, splenic B cells, and splenic T cells and purified using the column-based RNeasy Mini Kit (QIAGEN), according to the manufacturer’s protocol.

DNase-treated RNA (50–200 ng) was reverse-transcribed to cDNA using the High-Capacity RNA-to-cDNA Kit (Thermo Fisher Scientific). Real-time PCR was performed using the Power SYBR Green PCR Master Mix (Applied Biosystems) and an ABI ViiA7 Real-Time PCR system (Applied Biosystems). The qPCR amplification conditions were as follows: 95°C for 10 minutes, followed by 50 cycles of 95°C for 15 seconds and 60°C for 1 minute. All gene expression levels were normalized against the corresponding levels of *Actb* (encoding β-actin).

The following primers were used to amplify each murine gene: *Actb* (Forward: 5′-CATTGACAGCACCTACTCATGCC-3′, Reverse: 5′-GATTCCATACCCAGGAAGG-3′), *TNF* (Forward: 5′-GGTGCCTATGTCTCAGCCTCTT-3′, Reverse: 5′-GCCATAGAACTGATGAGAGGGAG-3′), *NFKBIA* (IκBα) (Forward: 5′-GCCAGGAATTGCTGAGGCACTT-3′, Reverse: 5′-GTCTGCGTCAAGACTGCTACAC-3′), *IL6* (Forward: 5′-TACCACTTCACAAGTCGGAGGC-3′, Reverse: 5′-CTGCAAGTGCATCATCGTTGTTC-3′), *TNFAIP3* (A20) (Forward: 5′-AGCAAGTGCAGGAAAGCTGGCT-3′, Reverse: 5′-GCTTTCGCAGAGGCAGTAACAG-3′), *ICAM* (Forward: 5′-AAACCAGACCCTGGAACTGCAC-3′, Reverse: 5′-GCCTGGCATTTCAGAGTCTGCT-3′), *VCAM* (Forward: 5′-GCTATGAGGATGGAAGACTCTGG-3′, Reverse: 5′-ACTTGTGCAGCCACCTGAGATC-3′), *CCL2* (Forward: 5′-GCTACAAGAGGATCACCAGCAG-3′, Reverse: 5′-GTCTGGACCCATTCCTTCTTGG-3′), *IL1B* (Forward: 5′-TGGACCTTCCAGGATGAGGACA-3′, Reverse: 5′-GTTCATCTCGGAGCCTGTAGTG-3′), *IL10* (Forward: 5′-CGGGAAGACAATAACTGCACCC-3′, Reverse: 5′-CGGTTAGCAGTATGTTGTCCAGC-3′), *RNF31* (HOIP) (Forward: 5′-GCCCTGAGGTGGGATTCTG-3′, Reverse: 5′-TTGAGGTAGTTTCGAGGCTCC-3′), *TLR7* (Forward: 5′-TGGCTCCCTTCTCAGGATGA-3′, Reverse: 5′-CCGTGTCCACATCGAAAACA-3′), *IP-10* (Forward: 5′-GCCGTCATTTTCTGCCTCAT-3′, Reverse: 5′-GCTTCCCTATGGCCCTCATT-3′), *IFNA4* (Forward: 5′-CCTGTGTGATGCAGGAACC-3′, Reverse: 5′-TCACCTCCCAGGCACAGA-3′), *IFNB1* (Forward: 5′-ATGAGTGGTGGTTGCAGGC-3′, Reverse: 5′-TGACCTTTCAAATGCAGTAGATTC-3′), *MX1* (Forward: 5′-AAAAACCTGGATCGGAACCAA-3′, Reverse: 5′-CGGGTCAACTTCACATTCAAAG-3′), *MX2* (Forward: 5′-TGGGCATTTGAAAAGCAGTAT-3′, Reverse: 5′-AGTGACCGTGTGCAGCATTTC-3′), *IFNG* (Forward: 5′-TGAACGCTACACACTGCATCTTGG-3′, Reverse: 5′-CGACTCCTTTTCCGCTTCCTGAG-3′), *IRF5* (Forward: 5′-GGTCAACGGGGAAAAGAAACT-3′, Reverse: 5′-CATCCACCCCTTCAGTGTACT-3′), *IRF7* (Forward: 5′-GAGACTGGCTATTGGGGGAG-3′, Reverse: 5′-GACCGAAATGCTTCCAGGG-3′), *IL18* (Forward: 5′-GACTCTTGCGTCAACTTCAAGG-3′, Reverse: 5′-CAGGCTGTCTTTTGTCAACGA-3′), *IL12 p35* (Forward: 5′-GAGGACTTGAAGATGTACCAG-3′, Reverse: 5′-CTATCTGTGTGAGGAGGGC-3′), *IL12 p40* (Forward: 5′-GACCCTGCCCATTGAACTGGC-3′, Reverse: 5′-CAACGTTGCATCCTAGGATCG-3′), *CCL1* (Forward: 5′-GGCTGCCGTGTGGATACAG-3′, Reverse: 5′-AGGTGATTTTGAACCCACGTTT-3′), and *IL4* (Forward: 5′-ATCATCGGCATTTTGAACGAGGTC-3′, Reverse: 5′-ACCTTGGAAGCCCTACAGACGA-3′).

The following primers were used to amplify each human gene: *Actb* (Forward: 5′-CACCATTGGCAATGAGCGGTTC-3′, Reverse: 5′-AGGTCTTTGCGGATGTCCACGT-3′), *IL6* (Forward: 5′-AGACAGCCACTCACCTCTTCAG-3′, Reverse: 5′- TTCTGCCAGTGCCTCTTTGCTG-3′), *TNFa* (Forward: 5′-CTCTTCTGCCTGCTGC ACTTTG-3′, Reverse: 5′-ATGGGCTACAGGCTTGTCACTC-3′), and *A20* (Forward: 5′-CTCAACTGGTGTCGAGAAGTCC-3′, Reverse: 5′-TTCCTTGAGCGTGCTGAACAGC-3′).

### Immunoblotting.

Cells were lysed with lysis buffer containing 50 mM Tris-HCl (pH 7.5), 150 mM NaCl, 1% Triton X-100, 2 mM phenylmethylsulfonyl fluoride, and protease inhibitor cocktail (MilliporeSigma) with or without 10 mM N-ethylmaleimide. For lysates of mouse tissues, 50 mg samples were homogenized and lysed with the lysis buffer described above using a tissue homogenizer (TissueRuptor, QIAGEN). Lysates were clarified by centrifugation at 20,400*g* for 20 minutes at 4°C. Samples (30 mg) were separated by SDS-PAGE and transferred to polyvinylidene difluoride membranes. After blocking in Tris-buffered saline containing 0.1% Tween 20 and 5% (w/v) nonfat dry milk for 90 minutes at room temperature, the membranes were incubated at 4°C overnight with the appropriate primary antibodies, followed by secondary antibodies for 90 minutes at room temperature. Membranes were visualized using enhanced chemiluminescence and analyzed on an LAS4000mini or LAS3000 instrument (GE Healthcare, now Cytiva).

The following antibodies were used for immunoblotting: anti-mouse IgG, HRP-linked (7076, Cell Signaling Technology); anti-IκBα (4812, Cell Signaling Technology); anti-pIκBα (9246, Cell Signaling Technology); anti-SHARPIN (4444, Cell Signaling Technology); anti–caspase-3 (9662, Cell Signaling Technology); anti–phospho-p44/42 MAPK (Erk1/2) (9101, Cell Signaling Technology); anti-Myc (4A6, MilliporeSigma); anti-HA (Tana2, Medical & Biological Laboratories, MBL); anti-DDDDK (PM020, MBL); anti–β-actin (A5316, MilliporeSigma); anti-tubulin (CLT9002, CEDARLANE); anti-SQSTM1/p62 (018-22141, Wako); HRP-conjugated anti-rabbit secondary antibody (NA934V, Amersham Pharmacia); AffiniPure Goat Anti-Mouse IgG, light chain-specific (115-005-174, Jackson Laboratory); goat anti-rabbit IgG (H+L) Highly Cross-Adsorbed Secondary Antibody, Alexa Fluor 488–conjugated (A-11034, Thermo Fisher Scientific); anti-linear ubiquitin chains (LUB9, in-house); anti–HOIL-1L (2E2, in-house); anti–HOIL-1L N-terminal (in-house); anti-SHARPIN (in-house); anti-HOIP (1CB2, in-house); and anti-Myc (9E10, in-house).

### ELISA.

To measure total immunoglobulins, anti-mouse IgM (1020-01), IgA (1040-01), IgG1 (1070-01), IgG2a (1080-01), IgG2b (1090-01), and IgG3 (1100-01) antibodies (SouthernBiotech; all at 1 mg/mL) were added to 96-well ELISA plates (Nunc MaxiSorp) and incubated at 4°C overnight. The wells were then blocked with 1% BSA in PBS at 4°C overnight. Appropriately diluted serum was incubated at room temperature for 60 minutes, and then HRP-conjugated anti-mouse IgM (1020-05), IgA (1040-05), IgG1 (1070-05), IgG2a (1080-05), IgG2b (1090-05), and IgG3 (1100-05) antibodies (SouthernBiotech) were added. OptEIA (BD Biosciences) was used as the substrate, and absorbance at 450 nm was measured using a microplate reader (Molecular Devices). Autoantibodies against SS-A (Alpha Diagnostics International, 5710), SS-B (Alpha Diagnostics International, 5810), ANA (Alpha Diagnostics International, 5210), and dsDNA (Alpha Diagnostics International, 5110) in serum were measured using the ELISA kits described, according to the manufacturer’s protocol.

### Hep-2 ANA test.

ANA were measured using a Hep-2 ANA test (4450, MBL). Serum was diluted 1:40 for the Hep-2 slides and stained as described by the manufacturer using donkey anti-mouse IgG Alexa Fluor 488 (A-11034, Thermo Fisher Scientific) as the secondary antibody. Confocal images were acquired under an Olympus FLUOVIEW FV1000 confocal laser scanning microscope. Image processing was carried out using ImageJ software (NIH).

### Flow cytometry analysis.

Single-cell suspensions were prepared from the spleen, peripheral blood, BMDMs, and splenic B and T cells of mice with the indicated genotypes. Cell Fc receptors were blocked using a purified anti-mouse CD16/32 antibody (101302, BioLegend) and then stained for 30 minutes at 4°C in the dark with the fluorochrome-conjugated antibodies described below. All samples were acquired on a FACSCanto II (BD Biosciences), and the results were analyzed with FlowJo software (Tree Star).

For FACS analyses, cells were stained with the following antibodies: biotin anti-mouse CD11b (79749, BioLegend), biotin anti-mouse CD21/CD35 (CR2/CR1) (123405, BioLegend), PE/Cy7 anti-mouse CD3ε (145-2C11, BioLegend), PE/Cy7 anti-mouse CD19 (115520, BioLegend), PE/Cy7 anti-mouse CD86 (105013, BioLegend), PE-conjugated anti-mouse CD80 (B7-1) (12-0801-81, eBioscience), APC anti-mouse F4/80 (123115, BioLegend), APC anti-mouse CD19 (152410, BioLegend), APC anti-mouse CD317 (BST2, PDCA-1) (127015, BioLegend), APC anti-mouse CD62L (104411, BioLegend), APC anti-mouse CD69 (104513, BioLegend), APC anti-mouse CD45R/B220 (103212, BioLegend), APC anti-mouse CD93 (AA4.1, early B lineage) (136510, BioLegend), APC/Fire 750 anti-mouse Ly-6G (127651, BioLegend), PE anti-mouse/human CD44 (103008, BioLegend), PE anti-mouse CD8a (100708, BioLegend), PE anti-mouse CD11c (American Hamster IgG) (117307, BioLegend), PE hamster anti-mouse CD95 (554258, BD Biosciences), anti-mouse CD23 PE (12-0232-82, eBioscience), PerCP/Cy5.5 anti-mouse CD4 (100434, BioLegend), Streptavidin-PerCP (405213, BioLegend), PerCP anti-mouse/human CD45R/B220 (103234, BioLegend), BV421 anti-mouse CD45 (563890, BD Biosciences), BV421 rat anti-mouse CD45R/B220 (562922, BD Biosciences), BV421 anti-mouse CD138 (Syndecan-1) (142507, BioLegend), BV421 anti-mouse/human CD11b (101235, BioLegend), FITC anti-mouse Ly-6G (127605, BioLegend), FITC anti-mouse CD3ε (100306, BioLegend), FITC anti-mouse CD38 (Rat IgG2a, k) (102705, BioLegend), FITC anti-mouse I-A/I-E (107606, BioLegend), FITC anti-mouse CD11b (553310, BD Biosciences), FITC rat anti-mouse IgM (553437, BD Biosciences), streptavidin APC (17-4317-82, eBioscience), PNA-bio (B-1075, Vector Laboratories), and biotin anti-mouse CD11c (American Hamster IgG) (117304, BioLegend).

### Histological scoring.

Histological scoring (Figure 3, F and G) of H&E-stained lung sections was conducted under an objective 4×/0.16 (UPlanApo) lens. Liver and stomach sections were observed under an objective 10×/0.40 (UPlanApo) lens, and adipose tissue sections were observed under an objective 20×/0.40 (UPlanApo) lens. Inflammatory cell infiltration was assessed in 10 microscopic fields using the following ordinal grading scale: 0 = absent; 1 = mild; 2 = moderate; 3 = marked; 4 = severe (18, 61). The average cellular infiltration scores were calculated for each mouse. H&E staining images were collected using cellSens standard software (Olympus).

### Isolation, culture, and stimulation of BMDMs and splenic B and T cells.

BMDMs were prepared as described previously (33). Briefly, bone marrow cells were isolated from the humerus, femurs, and tibias of mice of the indicated genotype. After removing reticulocytes, the residual cells were cultured for 6–7 days in RPMI 1640 medium containing 10% FBS, 100 IU/mL penicillin, 100 mg/mL streptomycin, 50 μM 2-mercaptoethanol (2-ME), and recombinant M-CSF (20 ng/mL; BioLegend; 576406). Floating cells were removed, and the adherent cells were used as BMDMs. The attached cells were seeded in 6-well plates and stimulated with LPS (*Escherichia coli* 055: B5; MilliporeSigma, 10 ng/mL) or R848 (catalog tlrl-r848-5; InvivoGen, 1 mg/mL) for the indicated times.

Splenic B cells were positively selected using anti-CD19 MicroBeads (Miltenyi Biotec) and a MACS Separation Column (Miltenyi Biotec). Purified splenic B cells were cultured in DMEM supplemented with 10% FBS, 50 μM 2-ME, 10 mM HEPES-KOH (pH 7.4), 100 IU/mL penicillin, and 100 mg/mL streptomycin and then stimulated with LPS, CpG-DNA (catalog tlrl-1826; InvivoGen, 100 nM), or R848 (catalog tlrl-r848-5; InvivoGen, 1 mg/mL) for the indicated times.

Based on negative selection of splenocytes on anti-CD19 MicroBeads (Miltenyi Biotec), splenic T cells were positively selected using anti-CD 90.2 MicroBeads (Miltenyi Biotec). Purified splenic T cells were cultured in DMEM supplemented with 10% FBS, 50 μM 2-ME, 10 mM HEPES-KOH (pH 7.4), 100 IU/mL penicillin, and 100 mg/mL streptomycin. Finally, 1 × 10^6^ T cells were stimulated with a combination of an anti-CD3ε antibody (1 mg/mL) and a soluble anti-CD28 antibody (1 mg/mL).

### DHR assay.

The data in Supplemental Figure 4, J and K, were generated using a neutrophil respiratory burst assay kit (601130, Cayman Chemical) to investigate ROS generation, according to the manufacturer’s protocol (31). Briefly, mouse peripheral blood was subjected to erythrocyte lysis and loading with DHR at 37°C for 15 minutes, followed by stimulation (or not) with PMA (100 nM or 200 nM) at 37°C for 45 minutes. ROS production was detected in the neutrophil (CD11b^+^Ly6G^+^) fraction by flow cytometry analysis.

### Plasmids and reagents.

The cDNAs used in this study were described previously (13, 20). Mutants of HOIL-1L were generated by PCR. cDNAs were ligated into the appropriate epitope-tag sequences and then subcloned into pcDNA3.1 (Invitrogen), pMAL-c2x (New England Biolabs Japan), pMXs-IP (Cell Biolabs, Inc), pMXs-neo (Cell Biolabs, Inc), and pMXs-IRES-Bsr (in house) vectors.

### Cell culture, transfection, and retroviral expression.

MEFs were generated in-house, HEK293T cells were gifted by Eijiro Nakamura (Kyoto University), Plat-E was gifted by Toshio Kitamura (University of Tokyo, Bunkyō, Tokyo, Japan), GP2-293 packaging cells were purchased from Takara Bio, and THP-1 cells were gifted by Hisanori Umehara (Kyoto University). MEFs, HEK293T cells, Plat-E packaging cells, and GP2-293 packaging cells were grown in DMEM containing 10% FBS, 100 IU/mL penicillin, and 100 mg/mL streptomycin. THP-1 cells were grown in RPMI 1640 medium containing 10% FBS, 100 IU/mL penicillin, and 100 mg/mL streptomycin. Transfections were performed using Lipofectamine 2000 (Invitrogen). For retroviral expression, pMXs-IP, pMXs-neo, or pMXs-IRES-Bsr encoding LUBAC components was transfected into Plat-E packaging cells or GP2-293 packaging cells, as described previously (20). The resultant viruses were used to infect LUBAC-TKO cells or THP-1 cells. Stably transduced cells were selected using puromycin (P9620-10ml, MilliporeSigma), G-418 (09380-44, Nacalai Tesque), or blasticidin (ant-bl-1, InvivoGen).

### Luciferase assays.

HEK293T cells were transfected with pGL4.32 (Luc2p/NF-κB–RE/Hygro) and pGL4.74 (hRLuc/TK) (Promega), along with plasmids encoding WT or mutant LUBAC components. Twenty-four hours after transfection, cells were lysed, and luciferase activity was measured using the Dual-Luciferase reporter assay system (Promega) on a Lumat Luminometer (Berthold).

### In vitro ubiquitination assay.

MBP-HOIL-1L or Petit-SHARPIN, along with recombinant HOIL-1L (WT or mutant), were incubated with E1 (100 ng), UbcH7/UBE2L3 (400 ng), ubiquitin (5 mg), and 2 mM ATP at 37°C for the indicated periods in 20 mL of buffer containing 20 mM Tris-HCl (pH 7.5), 5 mM MgCl_2_, and 1 mM DTT. After incubation, the reactions were terminated by addition of SDS sample buffer and analyzed by SDS-PAGE followed by immunoblotting.

### Expression and purification of recombinant proteins.

MBP-hHOIL-1L (WT, C460A, and R464H) was expressed in *E*. *coli* BL21 cells (CodonPlus DE3 230280). MBP-fusion proteins were purified using amylose resin (New England BioLabs). Petit-SHARPIN, recombinant E1, and recombinant UbcH7/UBE2L3 were prepared and purified as described previously (20).

### Cell viability assay using real-time cellular analysis technology.

Cell viability was monitored continuously as an impedance-based cell index using the iCELLigence system (ACEA Bioscience). For each sample, 20,000 cells were plated onto an E-Plate L8. On the next day, the cells were treated with TNF-α (R&D Systems, 410-MT, 40 ng/mL), and the cell index was monitored continuously. Data were normalized against cell indices at the time of TNF-α treatment.

### Measurement of LDH release.

LDH release was measured using the Cytotox96 Non-Radioactive Cytotoxicity Assay kit (Promega). Briefly, cells were seeded on a 24-well plate at a density of 1 × 10^5^ cells per well and then treated with TNF-α (10 ng/mL) plus CHX (Calbiochem, 239764, 20 mg/mL). After culture for the indicated periods, the medium was collected, and LDH levels were determined by measuring absorbance at 490 nm on a SpectraMax M5 (Molecular Devices).

### Detection of variants in the RBR domain of human HOIL-1L.

The public gnomAD (36) and an in-house WGS data set consisting of 3,026 high-depth Japanese WGS samples (JEWEL 3K) (7) were examined to identify missense variants in the RBR domain of HOIL-1L, and information about these variants was extracted.

### Association between the R464H variant and SLE.

To examine the association between the R464H variant in HOIL-1L and SLE, GWAS results from 148,312 Japanese samples (2,535 SLE cases versus 145,777 controls) were extracted and imputed with a reference panel built from JEWEL 3K and the 1KGP data sets. Details of the GWAS data set and the association analyses were described in a previous study (7).

### Association between the R464H variant and SS.

To explore the potential association between the R464H variant in HOIL-1L and SS, microarray data derived from 626 Japanese cases (378 primary SS and 248 secondary SS, coexisting with other rheumatic diseases) were genotyped by the Illumina Infinium Omni Express Exome array in combination with 140,248 Japanese controls from the BBJ (7). Diagnosis of SS was based on relevant criteria (62, 63). Using the data set after standard quality control of the microarray data, local imputation was performed on the flanking region of HOIL-1L R464H (chr20:1–5,000,000) using the same reference panel used for SLE analysis. In brief, variant phasing was conducted by shapeit2 (64), and imputation was done by impute4 (https://jmarchini.org/software/#impute-4) using the default parameters. Logistic regression analysis was performed by PLINK v2.0 using the first 10 principal components and sex as covariates.

### Statistics.

Statistical analyses were performed using GraphPad Prism 8 version 8.4.0. Statistical significance was determined using a 2-tailed Student’s *t* test, 1-way ANOVA followed by Dunnett’s multiple comparison test, and 1-way ANOVA followed by Tukey’s multiple comparison test. The exact sample sizes (*n*) used to calculate the statistics are provided in the figure legends. No data points were excluded. *P* values less than 0.05 were considered significant. All experiments were reproduced at least twice, with similar results each time.

### Study approval.

This study was approved by the ethical committee of each institution, and all animal studies were approved by Animal Research Committee, Graduate School of Medicine, Kyoto University (approval number: Med Kyo 23521).

### Data availability.

All other data supporting the findings of this study are available from the corresponding author upon reasonable request or in the Supporting Data Values XLS file.

## Author contributions

YF and KI conceived and designed the project. YF performed most of the experiments except for those in Figure 4 and Supplemental Figure 4. KK performed the experiments for Figure 4 and Supplemental Figure 4. CT, XL, HS, YK, and TS contributed to GWAS data and performed human data analysis. TI, KO, and AM provided advice on the project. YF and KI wrote the manuscript with contributions from all other authors.

## Supplementary Material

Supplemental data

Supporting data values

## Figures and Tables

**Table 1:**
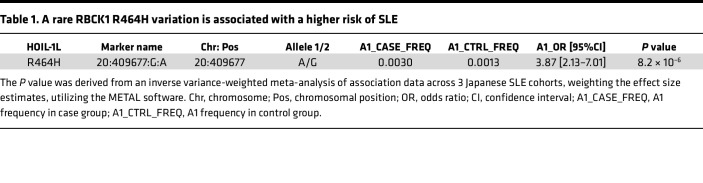
A rare RBCK1 R464H variation is associated with a higher risk of SLE

**Table 2:**
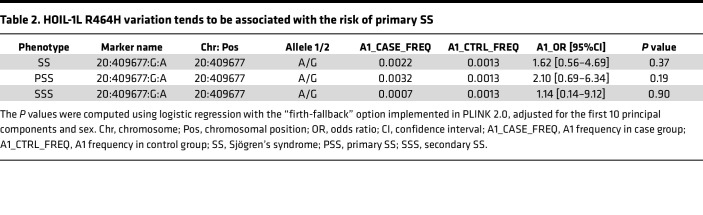
HOIL-1L R464H variation tends to be associated with the risk of primary SS

## Version 1 02/08/2024

Electronic publication
