# Supplementary material for: Attenuation of HOIL-1L ligase activity promotes systemic autoimmune disorders by augmenting linear ubiquitin signaling
Source: JCI Insight. 2024 Feb 8;9(3):e171108. doi: 10.1172/jci.insight.171108 (PMC10967397; doi:10.1172/jci.insight.171108)
Supplement: Supplemental data [file jciinsight-9-171108-s208.pdf]

Supplemental Material for:

Attenuation of HOIL-1L ligase activity promotes systemic autoimmune disorders by augmenting linear ubiquitin signaling

Authors:

Yasuhiro Fuseya, Keiichiro Kadoba, Xiaoxi Liu, Hiroyuki Suetsugu, Takeshi Iwasaki, Koichiro Ohmura, Takayuki Sumida, Yuta Kochi, Akio Morinobu, Chikashi Terao, & Kazuhiro Iwai*

*Corresponding author: Kazuhiro Iwai, Department of Molecular & Cellular Physiology Graduate School of Medicine, Kyoto University, Yoshida-konoe-cho, Sakyo-ku, Kyoto 606-8501, Japan. Tel: +81-75-753-4671, E-mail: kiwai@mcp.med.kyoto-u.ac.jp

This PDF file includes:

Supplemental Figure 1
Supplemental Figure 2
Supplemental Figure 3
Supplemental Figure 4
Supplemental Figure 5
Supplemental Figure 6

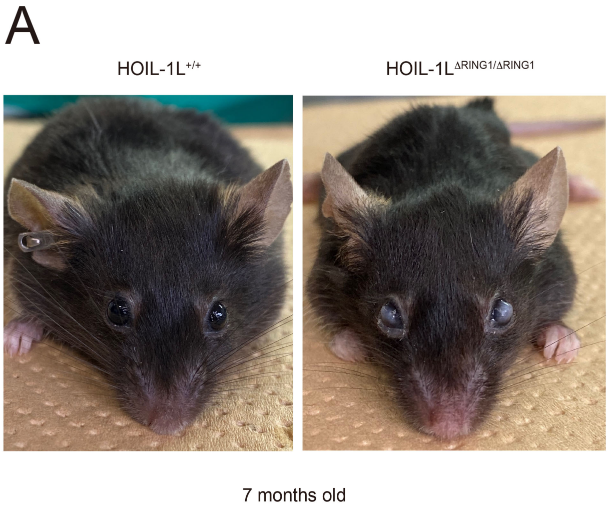

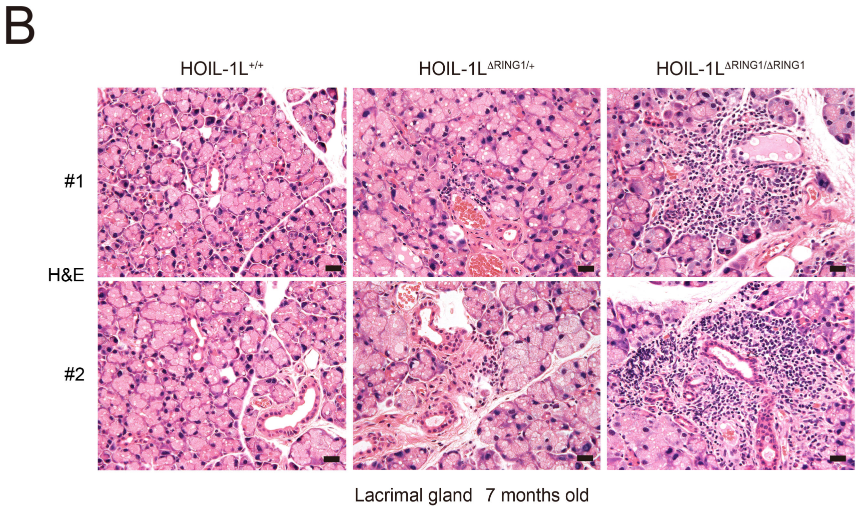

Fuseya et al. Supplemental Figure1

Supplemental Figure 1. HOIL-1L ΔRING1 mice show SS-like symptoms.

(A) Macroscopic pictures of female littermate mice of the indicated genotypes (7 months old). (**B)** Histological analysis (H&E staining) of lacrimal gland sections obtained from 7-month-old littermate mice of the indicated genotype. Scale bars, 20 μm.

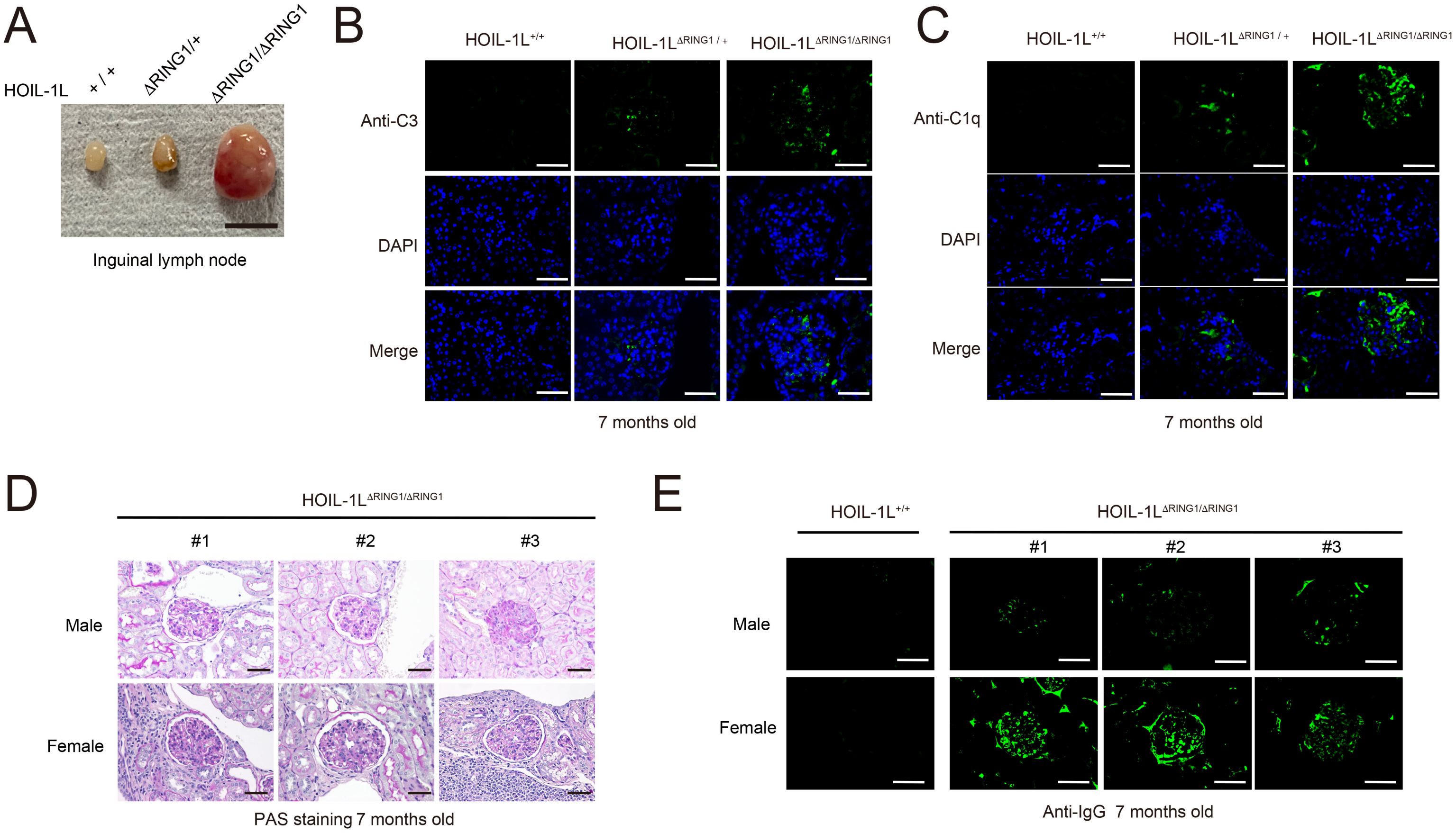

Fuseya et al. Supplemental Figure2

Supplemental Figure 2. HOIL-1L ΔRING1 mice developed lupus-like nephritis

(**A**) Macroscopic picture of the inguinal lymph nodes from the indicated female littermates (8 months old). Scale bars, 5 mm. (**B and C**) Histological analysis was performed by immunostaining kidney sections from 7-month-old littermate female mice of the indicated genotype to detect C3 (**B**) and C1q (**C**) (nuclei were stained with DAPI). Scale bars, 40 μm. (**D**) Histological analysis (PAS staining) of kidneys from 7-month-old female and male mice of the indicated genotype. Scale bars, 20 μm. n = 3 of each sex. (**E**) Immunostaining of kidney sections from 7-month-old mice of the indicated genotype to detect IgG. Scale bars, 50 μm.

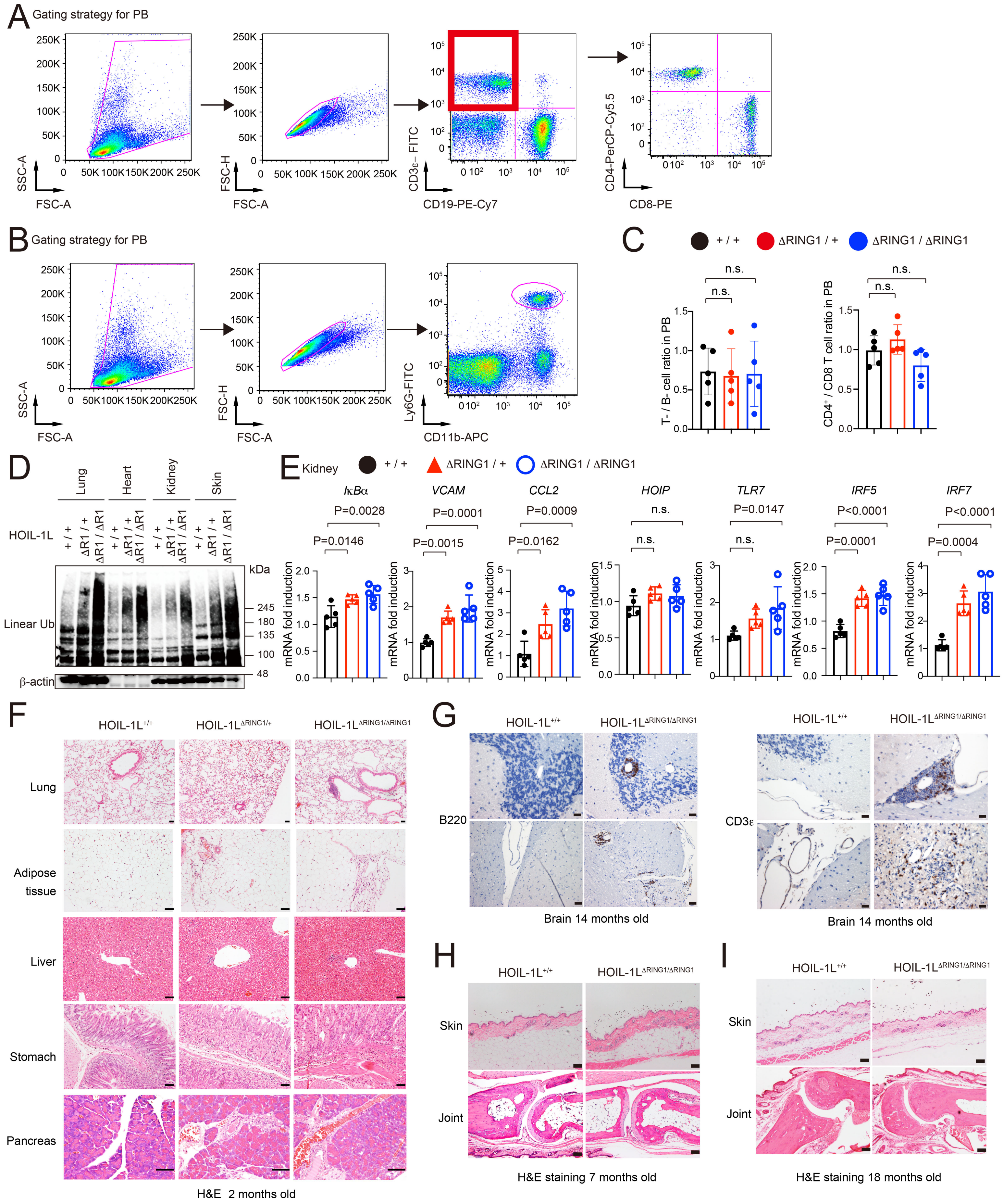

Fuseya et al. Supplemental Figure3

Supplemental Figure 3. HOIL-1LΔRING1 mice show characteristic features of SLE (**A** and **B**) Gating strategies used to identify lymphocytes (**A**) and neutrophils (**B**) in the peripheral blood (PB) of mice. (**C**) Quantification of leukocytes in the serum of 7–8-month-old female mice of the indicated genotype: T/B ratio and CD4/8 ratio. Data are expressed as the mean ± S.D. *P*-values were calculated by one-way ANOVA, followed by Dunnett's multiple comparisons test. n.s.: $p > 0.05$; n = 5 per group. (**D**) Lysates of organs from 7-month-old littermate female mice of the indicated genotype were subjected to immunoblotting to detect linear ubiquitin. (**E**) Quantitative PCR analysis of kidney tissue from 3–4-month-old female mice of the indicated genotype. Data are expressed as the mean ± S.D. *P*-values were calculated by one-way ANOVA, followed by Dunnett's multiple comparisons test. n.s.: $p > 0.05$. n = 5 per group. (**F**) H&E staining of multiple organs from 2-month-old female littermate mice of the indicated genotype. Scale bars, 50 μm. (**G**) Acquisition of macroscopic images, immunostaining for B220 and CD3ε in the brains of littermate (14-month-old) female mice of the indicated genotype. Scale bars, 50 μm. (**H and I**) Histological analysis, performed by H&E staining, of skin and joint sections from 7-month-old mice (**H**), and from 18-month-old mice (**I**). Littermate female mice of the indicated genotype. Scale bars, 100 μm (**H and I**).

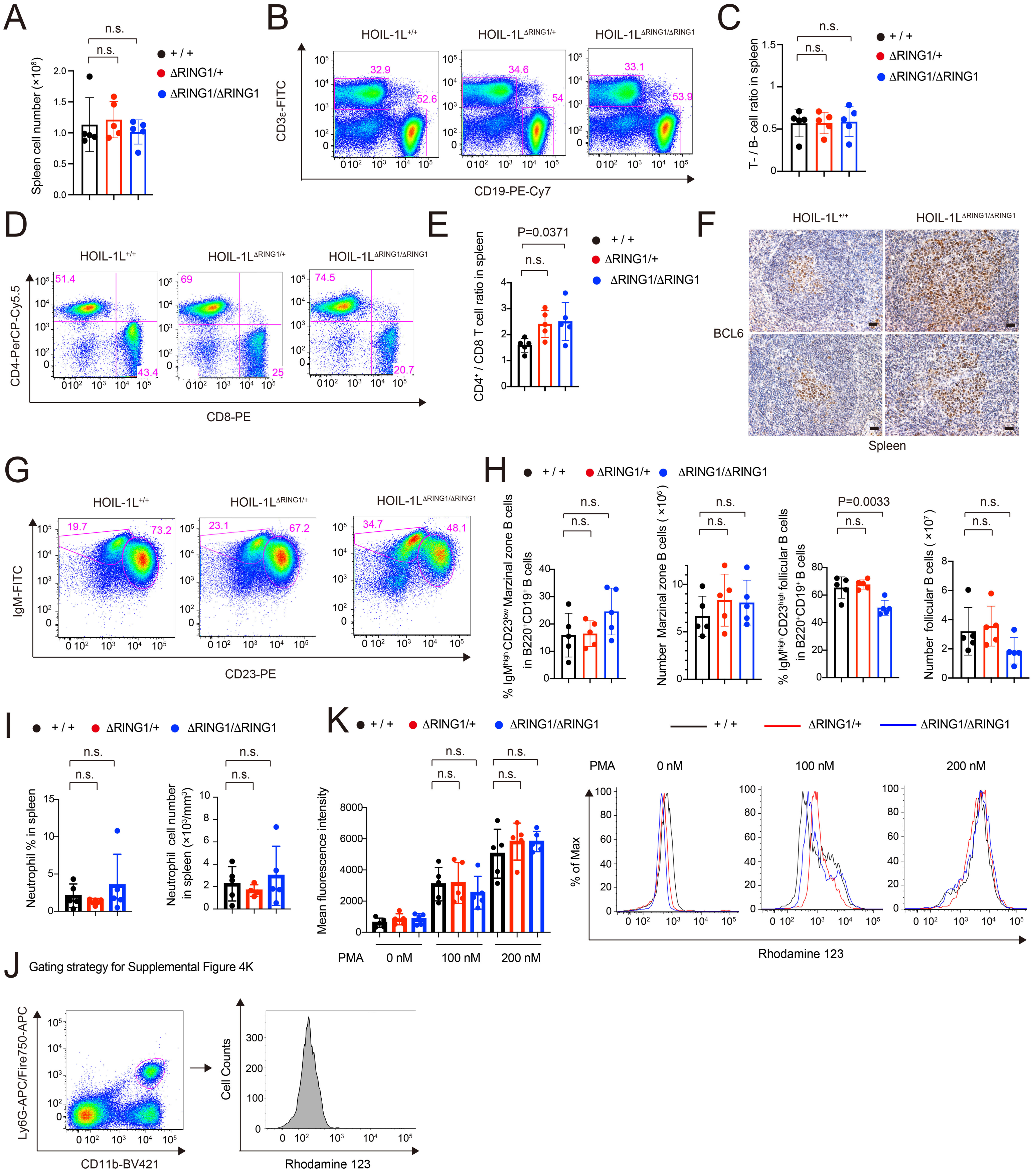

Fuseya et al. Supplemental Figure4

Supplemental Figure 4. Aged HOIL-1L ΔRING1 mice show activation of B and T lymphocytes.

(**A**) Cell counts in the spleen of 7–8-month-old female mice of the indicated genotype (n = 5 per group). (**B–E** and **G–K**) Representative flow cytometry plots, and quantification of splenocytes, from 7–8-month-old female mice of the indicated genotype. (**B** and **C**) T/B ratio, T cells ($CD3\varepsilon^{+}CD19^{-}$), and B cells ($CD3\varepsilon^{-}CD19^{+}$). (**D** and **E**) CD4/8 ratio within the T lymphocyte population. (**G and H**) Percentage and number of marginal zone B cells ($IgM^{high}CD23^{low}$) and follicular B cells ($IgM^{high}CD23^{high}$). (**I**) Percentage and number of splenic neutrophils. (**J**) Gating strategies used in Supplementary Figure 4K to identify neutrophils in peripheral blood. (**K**) Dihydrorhodamine (DHR) flow cytometry analysis of ROS production by neutrophils in peripheral blood of 7-month-old female mice of the indicated genotypes in the presence/absence of PMA (0 nM, 100 nM, and 200 nM). (**F**) Macroscopic images showing immunostaining for BCL6 in the spleen of littermate 7-month-old female mice of the indicated genotype. Scale bars, 20 µm. (**A**, **C,** **E**, **H, I** and **K**) Data are expressed as the mean ± S.D. (n = 4-5 per group). *P*-values were calculated by one-way ANOVA, followed by Dunnett's multiple comparisons test (**A**, **C,** **E**, **H and I**) and by Tukey's multiple comparisons test (**K**). n.s.: $p > 0.05$.

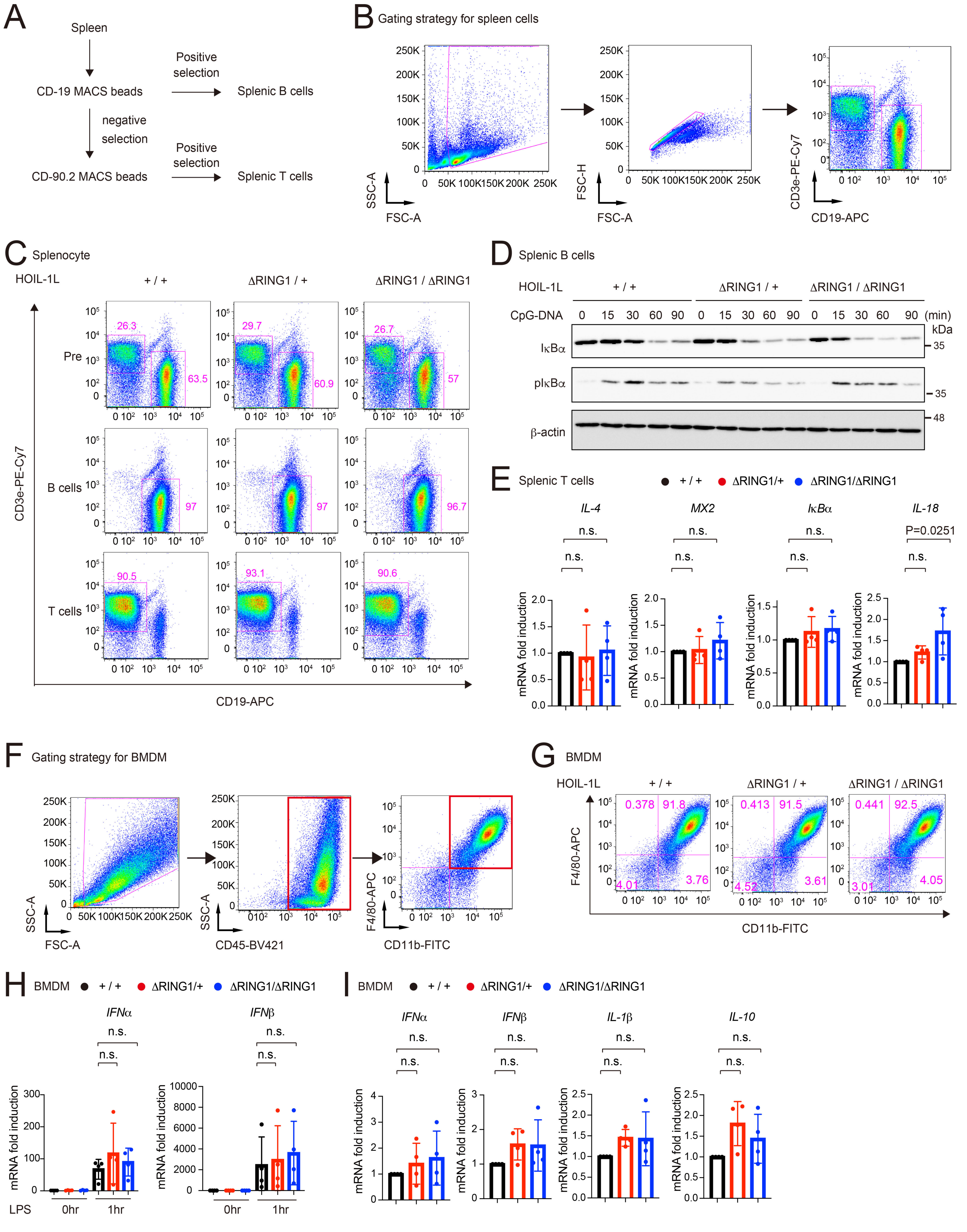

Fuseya et al. Supplemental Figure5

Supplemental Figure 5. Activation of signaling pathways in HOIL-1L ΔRING1 mice. (**A**) Flow chart showing isolation of primary B and T lymphocytes from the spleen by magnetic activated cell sorting (MACS). (**B**) Gating strategies used to identify B cells and T cells in the spleen. (**C**) Purity of splenic B and T lymphocytes isolated from the spleen of 12-week-old female littermates of the indicated genotype. Cells were isolated by MACS and analyzed by flow cytometry. (**D**) Lysates of primary splenic B cells isolated from 12-week-old female mice of the indicated genotype and stimulated with CpG-DNA (100 nM). Cells were probed as indicated. (**E**) Quantitative PCR analysis of splenic T lymphocytes isolated from 10–12-week-old female mice of the indicated genotype. Data are expressed as the mean (n = 4) ± S.D. (**F**) Gating strategies used to identify BMDMs. (**G**) Purity of BMDMs from 12-week-old female littermates of the indicated genotype, as analyzed by flow cytometry. (**H** and **I**) Quantitative PCR analysis of BMDMs from 10–12-week-old female mice of the indicated genotype. BMDMs were treated with or without LPS (10 ng ml^{-1}) for the indicated times, followed by qPCR (**H**). Data are expressed as the mean (n = 4) ± S.D. For each target, data sets of **H** (0 hr) and **I** are the same. (**E, H, and I**) *P*-values were calculated by one-way ANOVA, followed by Dunnett's multiple comparisons test. n.s.: $p > 0.05$.

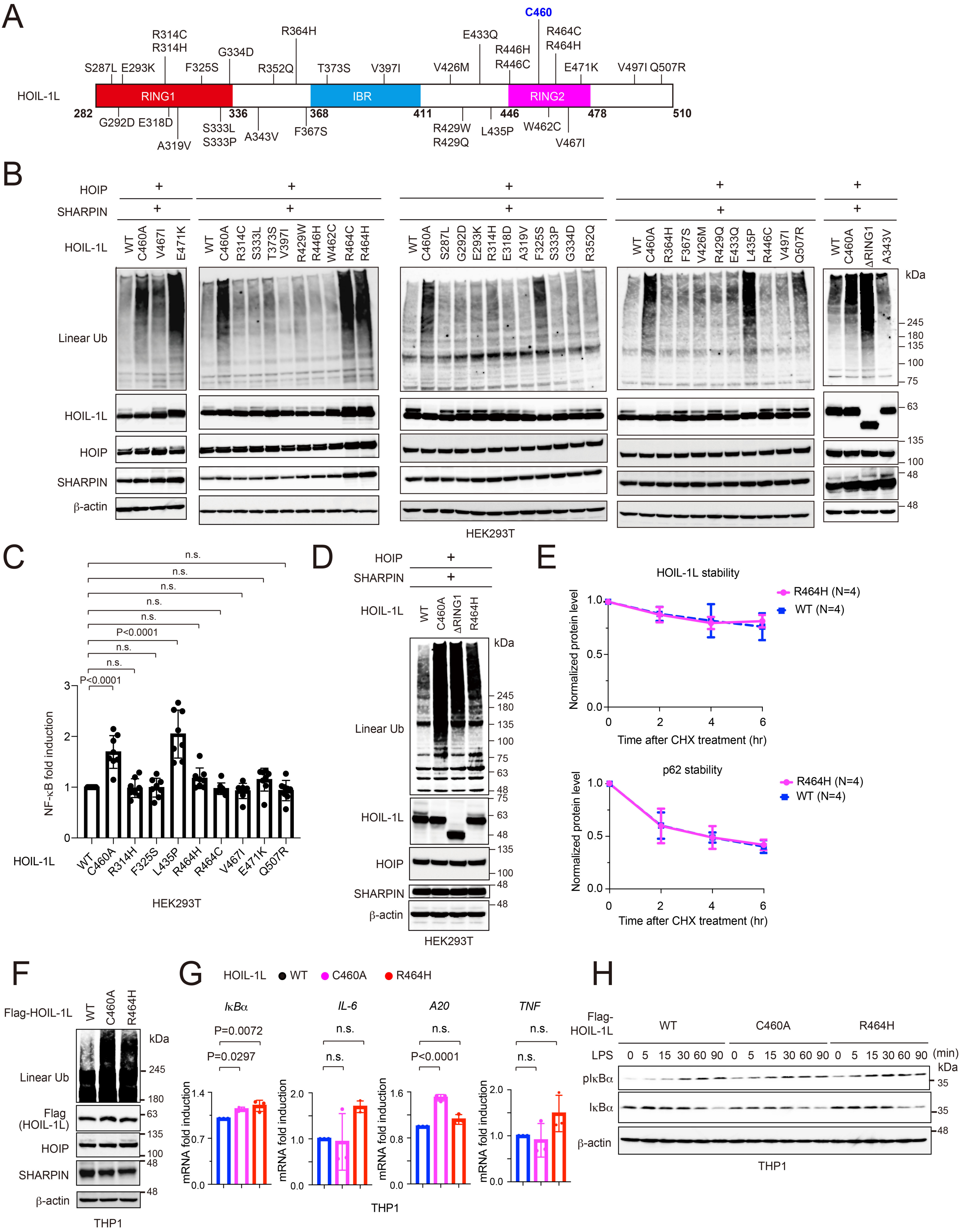

Fuseya et al. Supplemental Figure 6

Supplemental Figure 6. A HOIL-1L R464H variant is associated with a higher risk of SLE.

(**A**) Schematic representation of HOIL-1L, and the location of SNPs residing in the RING1-IBR-RING2 domains of HOIL-1L. (**B** and **D**) Expression of LUBAC components and the amounts of linear ubiquitin chains in HEK293T cells transfected with the indicated expression plasmids. (**C**) NF-κB activation in HEK293T cells transfected with the indicated expression plasmids and 5× NF-κB luciferase reporters was measured in a luciferase assay. Data are expressed as the mean (n = 8) ± S.D. *P*-values were calculated by one-way ANOVA, followed by Dunnett's multiple comparisons test. (**E**) Stability of HOIL-1L and p62 in LUBAC TKO MEFs expressing the indicated proteins and treated with CHX (20 μg ml^{-1}) for the indicated times. Data are expressed as the mean (n = 4) ± S.D. (**F**) Lysates from THP-1 cells stably expressing the indicated HOIL-1L proteins were probed as indicated. (**G**) QPCR analysis of THP-1 cells stably expressing the indicated HOIL-1L proteins. Mean values (n = 3) ± S.D. are shown, and *P*-values were calculated by one-way ANOVA, followed by Dunnett's multiple comparisons test. (**H**) THP-1 cells stably expressing the indicated HOIL-1L proteins were treated with LPS (20 μg ml^{-1}) for the indicated times and then assessed by immunoblotting with the indicated antibodies. (**C** and **G**) n.s.: $p > 0.05$.
